# Advanced paternal age as a risk factor for neurodevelopmental disorders: a translational study

**DOI:** 10.1186/s13229-020-00345-2

**Published:** 2020-06-23

**Authors:** Axel Krug, Markus Wöhr, Dominik Seffer, Henrike Rippberger, A. Özge Sungur, Bruno Dietsche, Frederike Stein, Sugirthan Sivalingam, Andreas J. Forstner, Stephanie H. Witt, Helene Dukal, Fabian Streit, Anna Maaser, Stefanie Heilmann-Heimbach, Till F. M. Andlauer, Stefan Herms, Per Hoffmann, Marcella Rietschel, Markus M. Nöthen, Martin Lackinger, Gerhard Schratt, Michael Koch, Rainer K. W. Schwarting, Tilo Kircher

**Affiliations:** 1 Department of Psychiatry and Psychotherapy, Philipps-University Marburg, 35039 Marburg, Germany; 2 Behavioral Neuroscience, Experimental and Biological Psychology, Philipps-University Marburg, Marburg, Germany; 3 Institute of Human Genetics, University of Bonn School of Medicine & University Hospital Bonn, Bonn, Germany; 4 Centre for Human Genetics, University of Marburg, Marburg, Germany; 5 Department of Psychiatry (UPK), University of Basel, Basel, Switzerland; 6 Department of Biomedicine, University of Basel, Basel, Switzerland; 7 Institute of Medical Genetics and Pathology, University Hospital Basel, Basel, Switzerland; 8 Department of Genetic Epidemiology in Psychiatry, Central Institute of Mental Health, Medical Faculty Mannheim/University of Heidelberg, Mannheim, Germany; 9 Max Planck Institute of Psychiatry, Munich, Germany; 10 Biochemisch-Pharmakologisches Centrum, Institut für Physiologische Chemie, Philipps-University Marburg, 35043 Marburg, Germany; 11 Lab of Systems Neuroscience, Department of Health Science and Technology, Institute for Neuroscience, Swiss Federal Institute of Technology, Zurich, Switzerland; 12 Department of Neuropharmacology, Brain Research Institute, Centre for Cognitive Sciences, University of Bremen, 28334 Bremen, Germany; 13 Center for Mind, Brain and Behavior, Marburg, Germany; 14 Department of Neurology, Klinikum rechts der Isar, School of Medicine, Technical University of Munich, Munich, Germany; 15 Laboratory for Behavioral Neuroscience, Department of Biology, Faculty of Science, University of Southern Denmark, Odense, Denmark; 16 Department of Psychiatry and Psychotherapy, University of Bonn, Bonn, Germany; 17 Department of Neurology, Klinikum rechts der Isar, School of Medicine, Technical University of Munich, Munich, Germany

**Keywords:** Advanced paternal age (APA), Diffusion tension imaging (DTI), Voxel-based morphometry (VBM), Social behavior, Ultrasonic vocalization

## Abstract

Advanced paternal age (APA) is a risk factor for several neurodevelopmental disorders, including autism and schizophrenia. The potential mechanisms conferring this risk are poorly understood. Here, we show that the personality traits schizotypy and neuroticism correlated with paternal age in healthy subjects (*N* = 677). Paternal age was further positively associated with gray matter volume (VBM, *N* = 342) in the right prefrontal and the right medial temporal cortex. The integrity of fiber tracts (DTI, *N* = 222) connecting these two areas correlated positively with paternal age. Genome-wide methylation analysis in humans showed differential methylation in APA individuals, linking APA to epigenetic mechanisms. A corresponding phenotype was obtained in our rat model. APA rats displayed social-communication deficits and emitted fewer pro-social ultrasonic vocalizations compared to controls. They further showed repetitive and stereotyped patterns of behavior, together with higher anxiety during early development. At the neurobiological level, microRNAs miR-132 and miR-134 were both differentially regulated in rats and humans depending on APA. This study demonstrates associations between APA and social behaviors across species. They might be driven by changes in the expression of microRNAs and/or epigenetic changes regulating neuronal plasticity, leading to brain morphological changes and fronto-hippocampal connectivity, a network which has been implicated in social interaction.

## Introduction

Autism spectrum disorder (ASD) and schizophrenia (SZ) are severe mental disorders with neurodevelopmental origin. They share substantial molecular genetic and early environmental risks [1–3]. An important environmental risk factor for ASD and SZ is advanced paternal age (APA) [4–8]. This is particularly relevant as ASD diagnoses have climbed steadily (Centers for Disease Control and Prevention estimates for 2000: 1 in 150; 2008: 1 in 88; 2014: 1 in 59) in the past couple of decades, along with a marked increase in the number of older fathers. For example, an increase of 10 years in paternal age was reported to be associated with a 21% higher risk of ASD [9].

ASD and SZ share elevated levels of schizotypal personality traits and neuroticism, which go along with difficulties in social life. In healthy subjects, these traits increase the risk for the disorders independent of other risks [10–13] and they are dimensionally distributed among healthy subjects and patients [14]. It is yet unknown, whether APA leads to neuroticism or schizotypy in the offspring, and if so, what the underlying mechanisms are.

As causes for the increased ASD and SZ risk of APA, de novo mutations [15, 16] and epigenetic reprogramming have been hypothesized. In rodent models of epigenetic modifications due to APA, DNA obtained from offspring of old fathers was hypermethylated [17], and multiple CpG sites spanning differentially methylated regions associated with brain-expressed imprinted genes were identified [18]. Moreover, Smith et al. [19] found a number of differentially expressed genes in the frontal cortex transcriptome obtained from offspring of old fathers, mainly in functional networks involved in inflammation and inflammatory disease. Epigenetic changes in the sperm of older males were detected [20], i.e., a significant loss of methylation, with similar brain DNA methylation abnormalities in offspring [21]. Importantly, APA in humans was also linked to epigenetic modifications in gene expression patterns [22–25], with DNA methylation, histone modifications, and alterations particularly in microRNAs (miRNAs) representing prime candidate mechanisms [26].

The expression levels of miRNAs are transmitted across generations, with alterations in miRNAs underlying the transgenerational effects of paternal lifetime experiences [27–29]. They represent particularly promising candidates for APA effects, since they have been repeatedly associated with ASD and SZ. Perturbations in miRNA expression have been described in post-mortem brain and peripheral blood samples from both ASD and SZ patients [30–32]. One of the most significant SZ-associated SNPs is located in the *MIR137* gene [33]. Further, based on results from rodent studies, miR-132 and miR-134 (as part of the miR379-410 cluster) appear to play a particularly important role in the context of neurodevelopmental disorders, since they regulate neuronal development and synaptic plasticity, as well as cognitive and social behavior [34]. Recently, miR-132 dysregulation was shown to trigger anxiety-related behavior [35], while deletion of the miR379-410 cluster in mice promoted anxiety and sociability [36]. Together, these findings in animal models support the notion that the increased risk of de novo mutations and epigenetic changes associated with ongoing cell divisions of spermatogonia in the testes throughout reproductive life underlie the association between APA and ASD or SZ.

To date, the current research raises the following problems and questions: (1) Is APA in humans related to personality traits, which in themselves increase the risk for ASD and SZ? (2) Although animal research has demonstrated effects of APA on cortical integrity, it is unclear whether APA has an effect on structural brain volumes in humans. (3) In humans, the psychosocial role of the father is difficult to disentangle from genetic effects and paternal and maternal age tend to be highly correlated. (4) Moreover, there is a lack of research regarding potential pathways conferring risk for psychiatric disorders, such as alterations in DNA methylation or miRNA regulation. Translational studies including animal models allow to manipulate the father’s age while controlling for mother’s age and to gain cell tissue from brain structures [37]. Given these unanswered questions, our present study had the following aims: First, the known link between APA and ASD as well as SZ led us to conduct a comprehensive set of behavioral phenotyping assays in APA rats, using well-established paradigms with high relevance and sensitivity for the diagnostic and associated symptoms [38]. Second, in a large human sample of healthy subjects, personality profiles as well as brain imaging phenotypes were investigated. It was hypothesized that paternal age is associated with disadvantageous personality traits regarding social and psychological functioning which may also be observed in rats. APA may also be related to a neural network implicated in social interaction. As we were interested in underlying mechanisms, we hypothesized that APA would lead to differential DNA methylation and miRNA regulation.

## Materials and methods

### Human study

The main cohort (*N* = 677) was recruited via public postings and e-mails sent to students of the RWTH Aachen and Marburg Universities, both in Germany. In Aachen, *N* = 380, in Marburg, *N* = 297 subjects were enrolled in the study. Personality traits in all subjects were assessed with the NEO-Five-factor-inventory (NEO-FFI) [39] and the Schizotypal Personality Questionnaire brief version (SPQ-B) [40] questionnaires; cognition was tested using an extensive battery (see supplementary materials). A randomly selected subsample of the main cohort was scanned with 3-T MRI using T1 sequences for voxel-based morphometry (VBM) analyses (*N* = 342). From the Marburg sample, diffusion tension imaging (DTI) data was acquired from *N* = 222 subjects. Characteristics of the behavioral, VBM, and DTI samples are given in Table 1. All subjects were screened for neuropsychiatric disorders (MINI or SCID) and subjects with any neuropsychiatric or neurological disorder were excluded from the study. For the analysis of the genome-wide methylation analysis, *N* = 21 male individuals were selected from our main cohort using the following criteria: (i) age < 30 years, (ii) non-smokers, (iii) no or little alcohol consumption, and (iv) having a young or old father. Eleven of these individuals (mean ± S.E.M. age 24.3 ± 2.5 years) had young fathers (mean paternal age 24.6 ± 1.6 years). The other ten individuals (mean age 23.8 ± 3.1 years) had old fathers (mean paternal age 43.7 ± 3.3 years). All individuals were of European origin. After a complete description of the procedure, subjects provided written informed consent to participate in the study. The protocol was approved by the local ethics committees of the medical faculty of the RWTH Aachen and Philipps-University Marburg according to the declaration of Helsinki. For a detailed description of methods, including statistical analyses, see supplementary materials.

**Table 1:** Characteristics of the human samples

Variable	Mean (SD)
**Main cohort (*N* = 677)**	
Sex ratio (men/women)	365/312
Age	26.02 (7.1)
Estimated verbal IQ	111.99 (12.24)
Years of education	15.87 (2.87)
Father’s age	31.34 (5.54)
Mother’s age	28.45 (4.87)
**Personality**	
SPQ-B	
Cognitive-perceptual deficits	1.31 (1.38)
Interpersonal deficits	2.02 (1.79)
Disorganization	1.22 (1.43)
SPQ-B total score	4.56 (3.36)
NEO-FFI	
Neuroticism	1.43 (.63)
Extraversion	2.26 (.51)
Openness	2.49 (.63)
Agreeableness	2.48 (.67)
Conscientiousness	2.59 (.58)
**Cognition**	
d2 test (KL, attention)	192.7 (37.8)
MWT-B (brief verbal IQ)	30.2 (3.3)
Trail-making-test	28.2 (12.6)
Semantic verbal fluency	25.9 (5.4)
Lexical verbal fluency	14.4 (5.1)
Letter-number-span	16.6 (2.6)
Symbol coding	66.2 (11.2)
Spatial span	18.9 (3.1)
VLMT	
Learning	58.6 (7.6)
Delayed recall	12.6 (2.3)
**VBM sample (*N* = 342)**	
Sex ratio (men/women)	198/144
Age	27.53 (7.90)
Estimated verbal IQ	115.27 (12.21)
Years of education	13.83 (2.73)
Father’s age	30.67 (5.27)
Mother’s age	28.09 (4.86)
**DTI sample (*N* = 222)**	
Sex ratio (men/women)	126/96
Age	26.11 (4.87)
Estimated verbal IQ	114.9 (11.82)
Father’s age	30.56 (5.53)
Mother’s age	28.08 (4.92)

### Animal study

Male and female Wistar rats (RccHan:WIST; Harlan, Venray, The Netherlands) served as subjects. Two cohorts of rats were tested. The first cohort was used for comprehensive longitudinal behavioral phenotyping. To this aim, female rats aged 2 months were bred with young male rats of 2 months (*N* = 10 females; control—CONT) or old male rats of 12 months (*N* = 10 females; advanced paternal age—APA). Breeding resulted in *N* = 16 litters, namely *N* = 9 CONT and *N* = 7 APA litters. Total progeny generated was *N* = 99 pups (female: 49; male: 50) under CONT conditions and *N* = 81 pups (female: 37; male: 44) under APA conditions (no APA effect on sex ratio was observed; *p* = .610). Average litter size for CONT litters was 11.00 ± 1.32 pups (females: 5.44 ± 0.77; males: 5.56 ± 1.13) and for APA litters 11.57 ± 0.84 pups (females: 5.29 ± 0.87; males: 6.29 ± 0.87). At day of birth (post-natal day (PND) 0), litters were culled to *N* = 8 pups per litter, with *N* = 4 females and *N* = 4 males whenever possible. In the APA condition, *N* = 2 male rats from different fathers had to be sacrificed due to teeth anomalies. Rats were exposed to a comprehensive set of behavioral phenotyping assays, including well-established paradigms with high relevance and sensitivity for the diagnostic and associated symptoms of ASD [38] and other neuropsychiatric disorders, such as anxiety and depression, but also SZ and bipolar disorder (BPD) [41–43]. A longitudinal design was applied. In the first 2 weeks of life, maternal care behavior (PND 2, 4, 6, 8, 10, and 12) was observed and isolation-induced 40-kHz ultrasonic vocalizations (USV) were assessed together with developmental milestones (PND 5, 7, 9, and 11). Shortly after weaning at PND 21, rough-and-tumble play behavior and pro-social 50-kHz USV (PND 32–34) were measured. Then, repetitive and stereotyped patterns of behavior (PND 64 ± 3) were assessed, followed by open field (PND 68–69 ± 3), elevated plus maze (PND 73 ± 3), and novel object recognition (PND 78–79 ± 3). Thereafter, half of the rats were trained in the spatial learning and reversal learning tasks (PND 114–123 ± 4), while the other half was exposed to the sucrose preference test (PND 114–123 ± 4). This was followed by the assessment of acoustic startle and pre-pulse inhibition (PPI) of acoustic startle (PND 188 ± 6), and amphetamine-induced hyperactivity and positive 50-kHz USV (PND 194–196 ± 6). Behavioral assays conducted after weaning were performed in male offspring due to higher ASD prevalence in males than females in humans, i.e., with *N* = 28 CONT male rats and *N* = 25 male APA rats. The second cohort was used for hippocampal neuronal plasticity and miRNA investigations. To this aim, female rats aged 2 months were bred with young male rats of 2 months (*N* = 10 females; control—CONT) or old male rats of 21 months (*N* = 10 females; advanced paternal age—APA). Breeding resulted in *N* = 17 litters, namely *N* = 9 CONT and *N* = 8 APA litters. Total progeny generated was *N* = 106 pups (female: 55; male: 51) under CONT conditions and *N* = 85 pups (female: 38; male: 47) under APA conditions (no APA effect on sex ratio was observed; *p* = .324). Average litter size for CONT litters was 10.60 ± 1.36 pups (females: 5.50 ± 0.70; males: 5.10 ± 0.82) and for APA litters 9.44 ± 1.20 pups (females: 4.22 ± 0.79; males: 5.22 ± 0.86). In the first 2 weeks of life, maternal care behavior (PND 2, 4, 6, 8, 10, and 12) was observed. Following weaning at PND 21, novel object recognition (PND 91–92 ± 5) was performed. Brains were taken out at PND 312 ± 5. For more details, see supplementary materials. All experimental procedures were performed according to the legal requirements of Germany and approved by the ethical committee of the local government (MR 20/35 Nr. 33/2012; Regierungspräsidium Gießen, Germany).

## Results

### Human Study

#### Behavioral data

Within the sample of *N* = 677 subjects, paternal age was linearly correlated with the sum score of the SPQ-B and all its subscales. Partial correlation coefficients were *r*_df=659_ = .126 for the sum total score (*p* = .001); *r*_df=659_ = .106 for “Cognitive perceptual deficits” (*p* = .006), *r*_df=659_ = .087 for “Interpersonal deficits” (*p* = .026), and *r*_df=659_ = .085 for “Disorganization” (*p* = .029). For the NEO-FFI, a significant partial correlation was found for neuroticism (*r*_df=659_ = .102; *p* = .008), and partial correlations with the other four traits were *r* < .065 (n.s.).

Maternal age also showed a nominally significant effect on SPQ-B scores, but not on NEO-FFI scores. For the SPQ-B, partial correlation coefficients were *r*_df=659_ = − .089, *p* = .022 for “Cognitive perceptual deficits,” *r*_df=659_ = − .076, *p* = .051 for “Interpersonal deficits,” *r*_df=659_=− .049, *p* = .206 for “Disorganization,” and *r*_df=659_ = − .098, *p* = .012 for the sum total score.

Regarding cognition, with the sole exception of semantic verbal fluency (partial *r* = − .118, *p* = .048), paternal age had no significant influence on any of the variables tested (all partial *r* < .048, all *p* > .128, n.s.). Maternal age also had no effect on cognition (all partial *r* < .085, all *p* > .154), except for the d2-attention test (*r* = − .079, *p* = .041). However, these two associations were not significant after correction for multiple testing.

#### VBM data

**Fig. 1:**
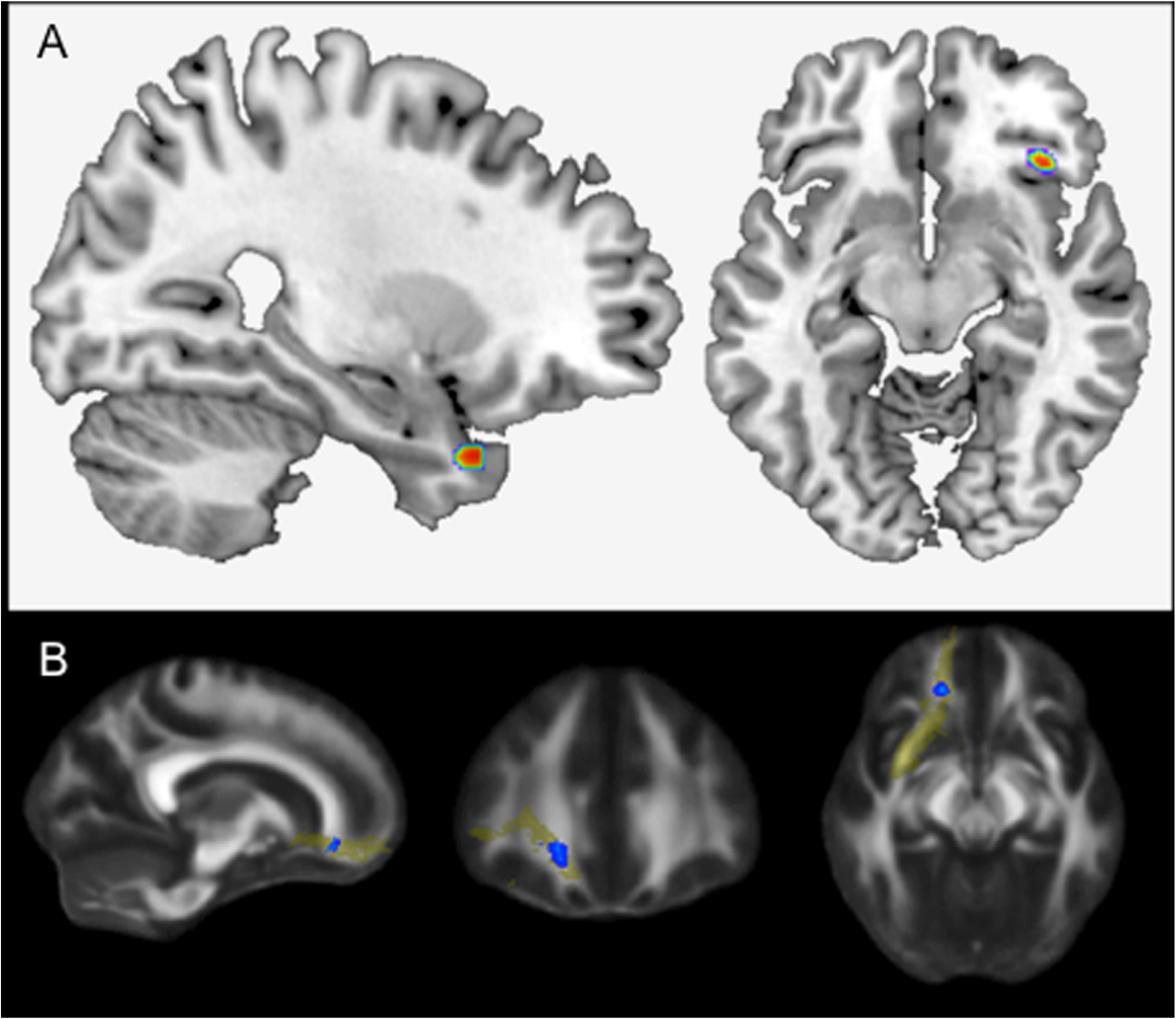
**a** Advanced paternal age is associated with increased right hippocampal volume (*N* = 342). Effects of paternal age (*p* < .001, uncorrected) on volume within the right hippocampal formation (cluster extent = 82 voxels) and right inferior frontal gyrus (cluster extent = 50 voxels). **b** Advanced paternal age is associated with white matter microstructure in the right uncinate fasciculus (*N* = 222). The effect of paternal age on the fractional anisotropy in the right uncinate fasciculus as a predefined region-of-interest (based on the John Hopkins University White-Matter Tractography Atlas)

In this sub-sample of *N* = 342, paternal age was linearly correlated with increases in gray matter in the right hippocampal formation (MNI coordinates xyz: 27; 15; − 33, cluster extent 82 voxels) as well as the right inferior frontal gyrus (IFG; MNI coordinates: 38; 27; − 9, cluster extent 50 voxels) (see Fig. 1a). Maternal age was linearly correlated with decreased gray matter volume in the right IFG (MNI coordinates: 38; 27; − 11, cluster extend 54 voxels, see Supplementary Fig. 1.

#### DTI data

In this sub-sample of *N* = 222, voxelwise analysis of the FA maps revealed a positive linear relationship between paternal age and white matter microstructure in the right uncinate fasciculus (UF; MNI coordinates xyz: 27; 15; − 33; *p* < 0.05, FWE-corrected; see Fig. 1b), connecting the frontal with the temporal lobe.

#### MicroRNAs

**Fig. 2:**
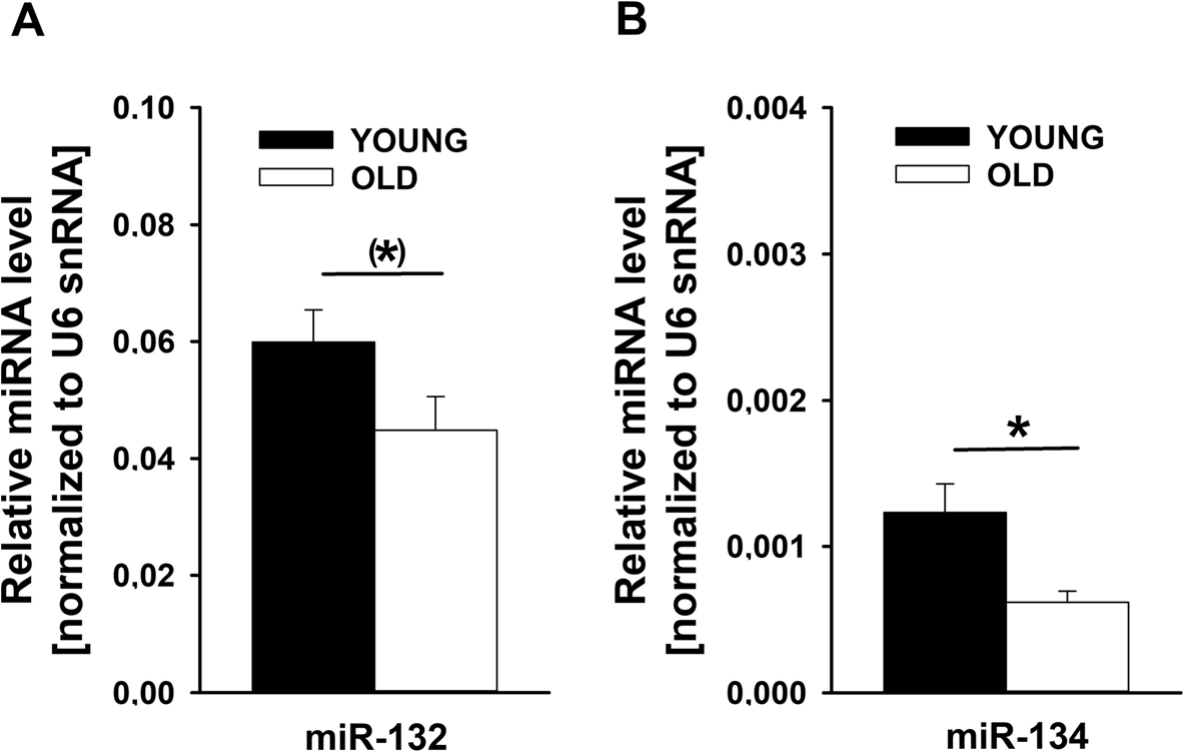
MicroRNA expression levels in human peripheral blood. **a** Analysis of miR-132 and **b** miR-134. Values represent mean transcript levels ± SEM relative to U6 small nuclear RNA (Rnu6). (*) = .056; *p < .01

In parallel to the animal study (see below), we measured the expression of mature miR-132 and miR-134 in human peripheral blood. The sample consisted of 18 male subjects with young (paternal age *m* = 22.7 ± 1.9) and 14 male subjects with old fathers (paternal age *m* = 38.2 ± 5.7). For miR-132, there was a marginally significant effect (*m* = .06 ± .02 for young fathers, *m* = .045 ± .02 for old fathers, *p* = .056); for miR-134, a significant effect was detected (*m* = .0012 ± .0007 for young fathers, *m* = .0006 ± .0003 for old fathers, *p* = .004) (see Fig. 2).

#### Methylation data

All *N* = 21 male individuals selected from our main cohort passed the sample-based quality controls and were included in the subsequent analyses. After probe-based quality control and normalization 426,125 CpG sites were tested for differential methylation using linear regression models.

The genome-wide methylation analysis of paternal age as a categorical trait did not show any significantly differentially methylated site after correction for multiple testing (Table 2). Three differentially methylated CpG sites had a nominal *p* value < 1 × 10^−6^, i.e., cg07350977 (*p*_corr_ = 0.07), cg12864235 (*p*_corr_ = 0.07), and cg13593090 (*p*_corr_ = 0.08). The first two of these CpG sites are located nearby the *CDH9* gene, which encodes for a type-II classic cadherin that mediates calcium-dependent cell-cell adhesion molecules [44]. The negative log fold-change (logFC < − 1.74) indicated a hypomethylation at both sites in individuals with young fathers. The third ranked CpG site cg13593090 is located near *ZNF266*, which encodes for the zinc finger protein 266 [45]. The positive log-fold change (logFC > 1.89) of this CpG site indicated a hypermethylation at this locus.

**Table 2:** Differentially methylated CpG sites with a nominal *p* value < 1 × 10^−5^

Probe ID	logFC	*p-*Value	Adj. *p* value	Nearby gene(s)	Region
cg07350977	− 1.60	3.04 × 10^−7^	0.07	*CDH9*	TSS200
cg12864235	− 1.74	3.17 × 10^−7^	0.07	*CDH9*	TSS200
cg13593090	1.90	5.32 × 10^−7^	0.08	*ZNF266*	TSS1500
cg02918054	1.30	5.14 × 10^−6^	0.47	*SSTR1*	5’UTR
cg12448161	− 1.60	5.51 × 10^−6^	0.47	*COL7A1*	TSS200
cg17744997	− 1.44	7.34 × 10^−6^	0.51	*–*	–
cg04158018	− 1.00	8.31 × 10^−6^	0.51	*NFE2*	TSS1500

CpG sites are ranked in descending order of statistical significance

*Probe ID* identifier from the Illumina CG database, *logFC* log fold-change, *Adj*. *p value p* value after Benjamini-Hochberg correction for multiple testing, *TSS* transcription start site, *UTR* untranslated region

### Animal study

#### Comprehensive longitudinal behavioral phenotyping

Although the associations obtained in the human part of the study are compelling, it is important to note that socio-economic and cultural factors coincide with APA. For instance, economic security, education level, and access to health services are all positively correlated with APA, possibly compensating to a certain extent the biological risk [46]. It was suggested that the association between APA and social deficits is primarily a function of selection factors, such that individuals with a genetic predisposition for schizotypal personality traits and thus being socially reclusive may not have the opportunity to conceive children until later in life [47, 48]. Consistently, paternal age was highly positively correlated with maternal age in the present human data set (*r* = .74, *p* < .001). These confounds highlight the importance of animal models when studying the impact of APA, since they allow to experimentally test causal relationships and to identify potential underlying mechanisms. We therefore bred female rats aged 2 months either with young male rats of 2 months (*N* = 10 females; CONT) or old male rats of 12 months (*N* = 10 females; APA). Offspring was then exposed to a comprehensive set of behavioral phenotyping assays, including well-established paradigms with high relevance and sensitivity for the diagnostic and associated symptoms of ASD [38] and other neuropsychiatric disorders, such as anxiety and depression, but also SZ and BPD [41–43].

**Fig. 3:**
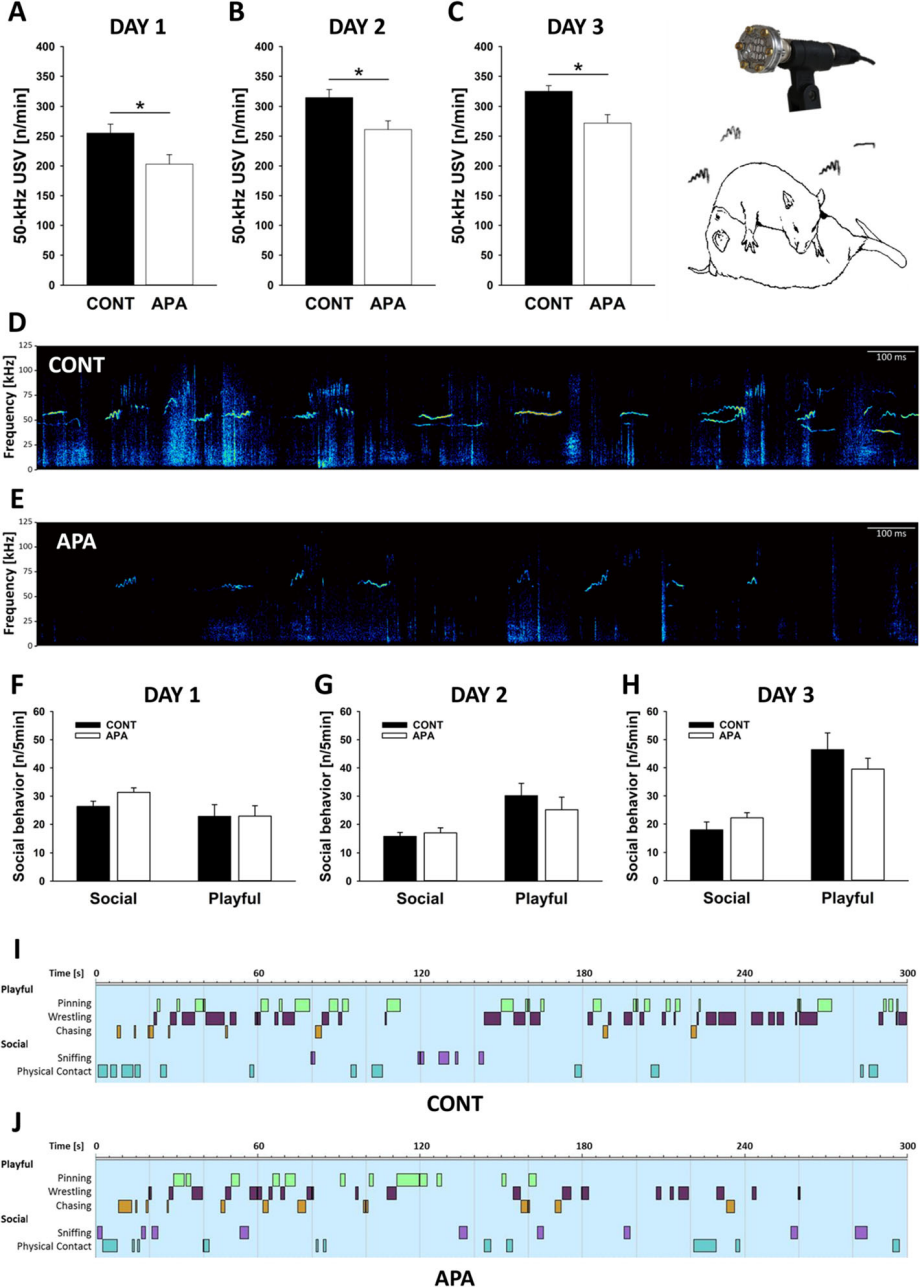
Deficits in ultrasonic communication during rough-and-tumble play in APA rats. **a**–**c** Emission of pro-social 50-kHz ultrasonic vocalizations (USV) [n/min] on the (**a**) first, (**b**) second, and (**c**) third testing day. **d**, **e** Exemplary spectrograms of pro-social 50-kHz USV emitted during the third testing day by a pair of (**d**) CONT and (**e**) APA rats. **f**–**h** Social and playful behavior [n/5min] on the (**f**) first, (**g**) second, and (**h**) third testing day. **i**, **j** Exemplary ethograms depicting frequency and duration of playful (namely pinning, wrestling, and chasing) and other social events (namely sniffing and physical contact) displayed on the third testing day by a pair of (**i**) CONT and (**j**) APA rats. *p < .05

By these means, we obtained evidence for an ASD-related behavioral phenotype in APA rats, including social-communication deficits, repetitive and stereotyped patterns of behavior, and elevated levels of anxiety-related behavior during early development, together with alterations in developmental milestones. Firstly, during rough-and-tumble play, APA rats displayed social-communication deficits and emitted fewer pro-social 50-kHz USV than CONT rats. This APA effect was consistently detectable on all three rough-and-tumble play days (*p* = .026, *p* = .011, and *p* = .005; respectively; see Fig. 3a–c; for exemplary spectrograms, see Fig. 3d, e). The frequency of engaging in rough-and-tumble play (*p* = .988, *p* = .433, and *p* = .341; respectively) and non-playful social interactions (*p* = .056, *p* = .573, and *p* = .218; respectively) did not differ between experimental groups (see Fig. 3f–h; for exemplary ethograms, see Fig. 3i, j). Likewise, the time engaged in playful and non-playful interactions did not differ (not shown).

**Fig. 4:**
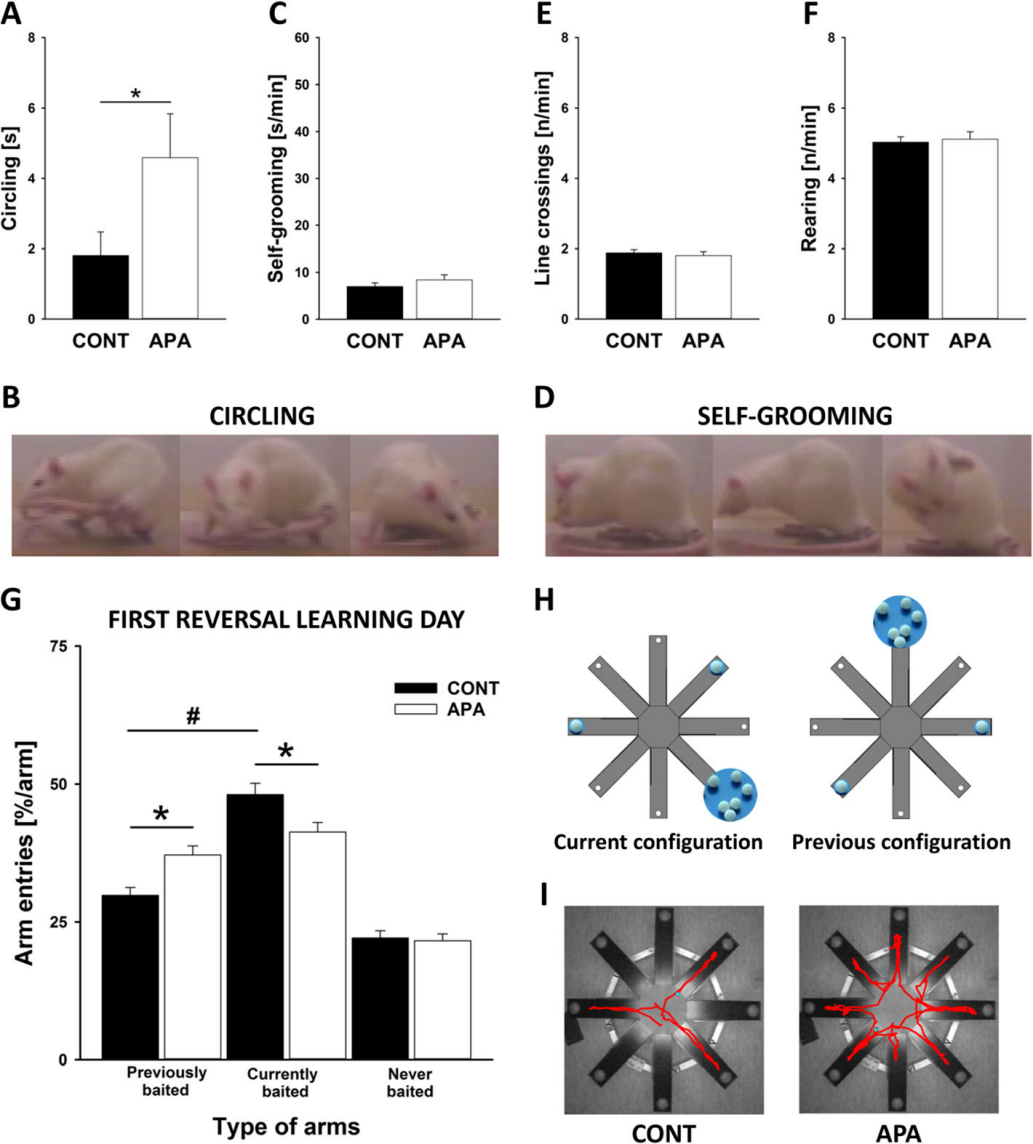
Repetitive and stereotyped patterns of behavior in APA rats. **a** Time spent circling [s] during tail-chasing. **b** Exemplary image of circling behavior. **c** Time spent self-grooming [s/min]. **d** Exemplary image of self-grooming behavior. **e** Frequency of line crossings [n/min]. **f** Frequency of rearing behavior [n/min]. **g** Repetitive and stereotyped behavior was also tested using a radial eight arm maze with three consistently baited arms. After this spatial learning period, subjects were tested for reversal learning and the positions of baited arms were changed. Arm entries [%/arm] in previously, currently, and never baited arms during all four sessions of the first reversal learning day were counted. **h** Schematic illustration of the different spatial learning and reversal learning configurations (termed previous and current configuration, respectively). **i** Exemplary tracking profiles of a single rat during the fourth trial of the first reversal learning day, measured by using the automated video tracking software EthoVision (Noldus Information Technology, Wageningen, The Netherlands), with tracking profiles shown in red. *p < .05; ^#^*p* < .05

Secondly, APA rats displayed repetitive and stereotyped patterns of behavior. Specifically, APA rats showed more circling behavior, i.e., tail-chasing than CONT rats (*p* = .048; see Fig. 4a; for exemplary images, see Fig. 4b), while self-grooming behavior was not different between groups (*p* = .275; see Fig. 4c; for exemplary images, see Fig. 4d). Locomotor activity, as assessed by distance travelled and rearing behavior, also did not differ between groups (*p* = .585 and *p* = .740, respectively; see Fig. 4e, f). Consistently, locomotor activity in the open field did not differ between CONT and APA rats, with distance travelled being similar in both groups on the first and second testing day (*p* = .753 and *p* = .786, respectively). Rearing behavior also did not differ on both testing days (*p* = .549 and *p* = .963, respectively). Importantly, repetitive and stereotyped patterns of behavior were not just detectable at the motor behavior level but also at a higher cognitive level. While CONT and APA rats displayed intact spatial learning abilities in a radial eight arm maze (for details, see below), APA rats made a high number of perseveration errors during reversal learning (Age × Arm: *p* = .003; see Fig. 4g; for a schematic illustration of the different spatial learning and reversal learning configurations, see Fig. 4h; for exemplary tracking profiles, see Fig. 4i). Specifically, during the first day of reversal learning, CONT rats entered the arms currently baited with food more often than the arms previously baited (*p* < .001). No such preference for currently baited over previously baited arms was seen in APA rats (*p* = .213). When comparing CONT and APA rats, APA rats entered previously baited arms more often than CONT rats (*p* = .002). The opposite was true for currently baited arms. Here, APA rats displayed fewer entries than CONT rats (*p* = .021). Arm entries in never baited arms did not differ between groups (*p* = .771). Together, this shows that repetitive and stereotyped patterns of behavior occurred more often in APA rats than in CONT rats, both at the motor behavior level but also at a higher cognitive level.

**Fig. 5:**
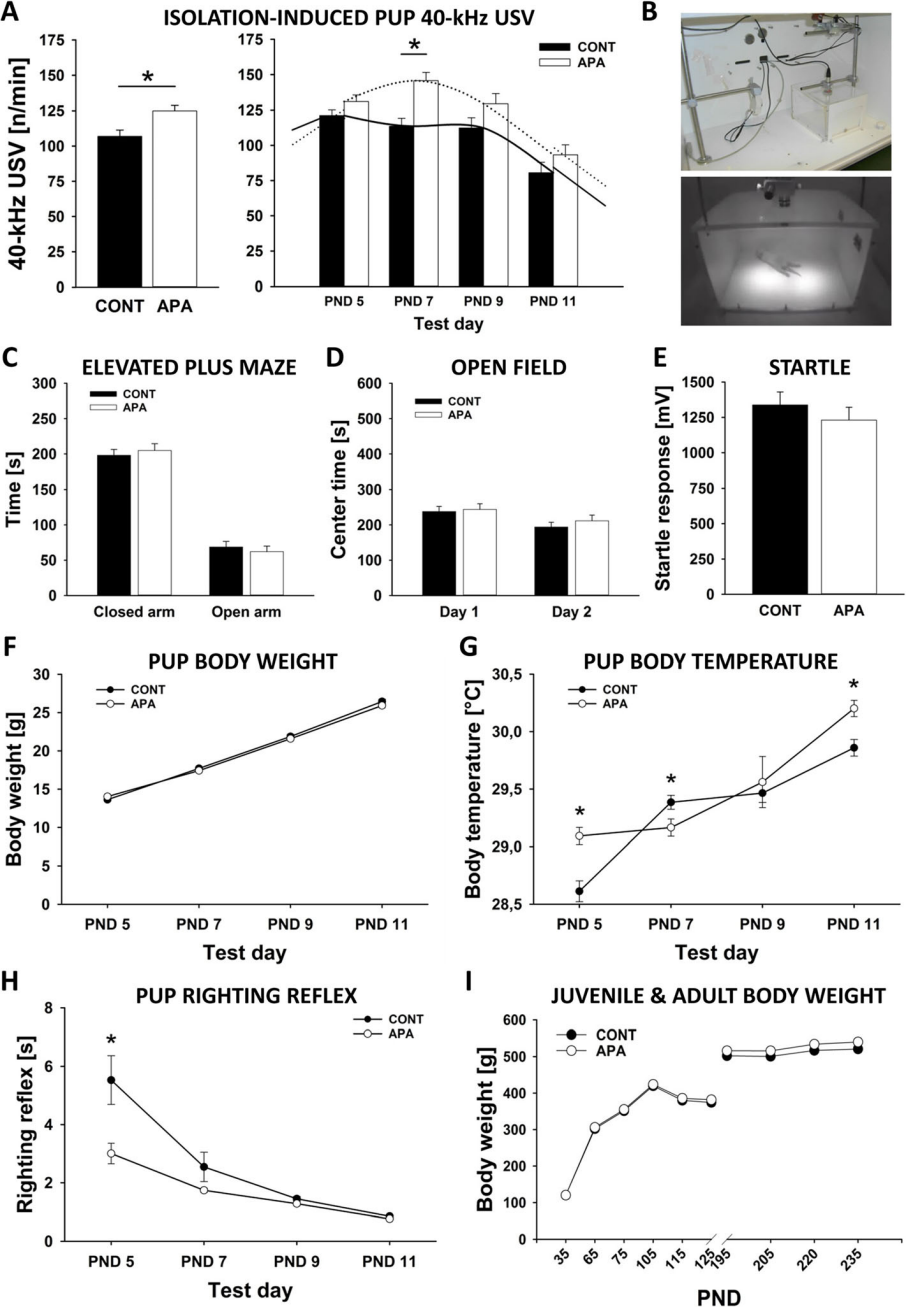
Higher levels of anxiety-related behavior during early development in APA rats. **a** Emission of isolation-induced 40-kHz ultrasonic vocalizations (USV) [n/min] averaged over the four testing days and shown separately for postnatal days (PND) 5, 7, 9, and 11. **b** Test chamber used for measuring isolation-induced 40-kHz USV. **c** Time spent on closed and open arms [s] in the elevated plus maze. **d** Time spent in the center [s] of the open field on two consecutive days. **e** Acoustic startle response [mV] measured during the habituation phase of the pre-pulse inhibition of acoustic startle test. **f** Pup body weight gain [g]. **g** Pup body temperature [°C] measured after the isolation period. **h** Pup righting reflex [s] after placing the pups on their back. **i** Juvenile and adult body weight gain [g]. *p < .050

In addition to social-communication deficits and repetitive and stereotyped patterns of behavior, APA rats displayed higher levels of anxiety-related behavior during early development. Specifically, emission of isolation-induced 40-kHz USV in the first 2 weeks of life was affected by APA, with USV emission being higher in APA than CONT rats (Age: *p* = .004, PND: *p* < .001, Age × PND: *p* = .156; see Fig. 5a, b). As expected, no sex differences were observed (Sex: *p* = .990, Age × Sex: *p* = .781, Sex × PND: *p* = .401, Age × Sex × PND: *p* = .781). Most prominent differences between CONT and APA rats were seen on PND 7 (*p* < .001), with minor differences on PND 5, 9, and 11 (*p* = .098, *p* = .092, and *p* = .222; respectively). In adulthood, no evidence for an effect of APA on anxiety-related behavior was obtained in the elevated plus maze. Specifically, CONT and APA rats did not differ in the time they spent on open arms (*p* = .559; see Fig. 5c) nor risk assessment behavior (*p* = .334). Locomotor activity, as assessed by means of total number of arm entries, was not significantly different between CONT and APA rats (*p* = .079). Latencies to enter open or closed arms also did not differ between groups (*p* = .277 and *p* = .461; receptively). Consistently, the time spent in the center of the open field also did not differ between groups on the first and second testing day (*p* = .784 and *p* = .395; respectively; see Fig. 5d). Moreover, startle responses did not differ between CONT and APA rats (*p* = .382; see Fig. 5e).

During early development, CONT and APA pups further differed in body weight gain (Age: *p* = .579, Sex: *p* = .009, Age × Sex: *p* = .749, PND: *p* < .001, Age × PND: *p* < .001, Sex × PND: *p* = .458, Age × Sex × PND: *p* = .805; see Fig. 5f) and temperature regulation (Age: *p* = .030, Sex: *p* = .5, Age × Sex: *p* = .834, PND: *p* < .001, Age × PND: *p* = .004, Sex × PND: *p* = .426, Age × Sex × PND: *p* = .403; see Fig. 5g). While body weight gain was delayed overall without resulting in clear differences at individual PNDs (all *p* values > .050), body temperature was higher in APA than CONT rats on PND 5 and PND 11 (*p* < .001 and *p* = .001; respectively) but lower on PND 7 (*p* = .023) and did not differ on PND 9 (*p* = .674). Moreover, evidence for differences in developmental milestones was obtained, with the righting reflex being more prominent in APA than CONT rats (Age: *p* = .002, Sex: *p* = .146, Age × Sex: *p* = .263, PND: *p* < .001, Age × PND: *p* = .004, Sex × PND: *p* = .525, Age × Sex × PND: *p* = .577; see Fig. 5h). It took APA rats less time to right themselves on all four paws after placing them on their back, particularly on PND 5 (*p* = .008), while on PND 7, 9, and 11 CONT rats were as fast as APA rats (*p* = .135, *p* = .109, and *p* = .075, respectively). After weaning, body weight did not differ between CONT and APA rats (all *p* values < .05; see Fig. 5i).

Importantly, no evidence for behavioral phenotypes with relevance for depression, nor other neurodevelopmental disorders, such as SZ or BPD, was obtained, highlighting the specificity of the observed behavioral alterations linked to ASD. Specifically, sucrose preference used to assess anhedonia-like alterations with relevance to depression was not affected by APA. In response to the two sucrose concentrations applied, both groups showed a clear preference for the higher 1.0% sucrose solution (CONT: *p* < .001; APA: *p* < .001; Supplementary Fig. 2a) and the lower 0.5% sucrose solution (CONT: *p* = .001; APA: *p* < .001; Supplementary Fig. 2b). Preferences were of similar magnitude and did not differ between CONT and APA rats (*p* = .667 and *p* = .925, respectively). Sucrose and water intake also did not differ between groups (all *p* values > .05).

Likewise, PPI of acoustic startle was not affected by APA. As expected, PPI magnitude was dependent on the intensity of the pre-pulse, yet independent from APA (AGE: *p* = .114; PRE_PULSE: *p* < .001; Age × PRE_PULSE: *p* = .277; Supplementary Fig. 2c). Both CONT and APA rats displayed reduced startle responses when pre-pulses of 64, 68, 72, and 76 dB were presented before pulses of 105 dB (all *p* values < .001) and the groups did not differ in PPI magnitude (*p* = .804, *p* = .07, *p* = .135, and *p* = .062; respectively).

When modeling mania-like elevated drive in rats exposed to amphetamine, CONT and APA rats displayed an increase in locomotor activity of similar strength. Locomotor activity under baseline and saline conditions did not differ between CONT and APA rats (*p* = .113 and *p* = .051, respectively; Supplementary Fig. 2d, e). In both groups, locomotor activity in response to amphetamine was higher than under baseline or saline conditions (CONT: *p* < .001 and *p* < .001, respectively; APA: *p* < .001 and *p* < .001, respectively), but did not differ from each other (*p* = .998; Supplementary Fig. 2f). Moreover, the emission of amphetamine-induced 50-kHz USV, possibly reflecting mania-like elevated mood [49, 50], did not differ between CONT and APA rats. While 50-kHz USV emission during baseline and saline conditions was minimal and not different between groups (*p* = .775 and *p* = .826, respectively; Supplementary Fig. 2g, h), a prominent increase in 50-kHz USV was seen following amphetamine administration, as compared to baseline and saline conditions (CONT: *p* < .001 and *p* < .001, respectively; APA: *p* < .001 and *p* < .001, respectively; Supplementary Fig. 2i). Increases were of similar strength in both groups, with CONT and APA rats not differing from each other in the emission of amphetamine-induced 50-kHz USV (*p* = .475), indicating that APA effects on 50-kHz USV production specifically occur in a social context, rough-and-tumble play, but not in a non-social one, namely amphetamine treatment. A more detailed analysis of 50-kHz USV subtypes further indicated that the typical increase in frequency-modulated 50-kHz USV, including STEP, TRILL, and MIXED subtypes, following amphetamine administration occurred in both groups with similar magnitude, as compared to baseline and saline conditions (CONT: *p* < .001 and *p* = .015; APA: *p* = .026 and *p* < .001). No differences in the occurrence of the different 50-kHz USV subtypes were evident between CONT and APA rats during baseline, saline, and amphetamine treatment (all *p* values > .05; Supplementary Fig. 2j).

Finally, no evidence for cognitive deficits was detected. In the novel object recognition paradigm, both CONT and APA rats displayed a clear preference for the novel object (*p* < .001 and *p* = .001, respectively), reflecting intact memory functioning. Novel object preference did not differ between groups (*p* = .673; Supplementary Fig. 3a). Notably, intact object memory recognition was also evident in the second cohort used for the assessment of hippocampal neuronal plasticity and miRNAs (not shown). Likewise, spatial learning abilities were intact, as reflected by a reduction in the proportion of wrong arm entries over training days in both groups (*p* = .733; Supplementary Fig. 3b). In the probe trial, CONT and APA rats displayed a clear preference for the arms previously baited with food (*p* = .02 and *p* = .035; respectively), with no difference between groups in the time spent in previously baited and not baited arms (*p* = .771 and *p* = .771; respectively; Supplementary Fig. 3c).

There is evidence that mothers adjust their investment in offspring growth and development depending on mate quality, resulting either in differential allocation or reproductive compensation [26], and that maternal care, in turn, affects emotion regulation and social development in the offspring, particularly the capacity to function adaptively within the social milieu [51]. It appears therefore possible that the observed APA effects are mediated by APA-dependent differences in maternal care. To test this possibility, we assessed maternal behavior every second day during the first 2 weeks of life. Continuous observations of maternal behavior were made, with *N* = 240 observations/mother/day, resulting in *N* = 1.440 observations per mother and a total observation duration of 24 h. Despite this rather extensive observation, no evidence for differences in maternal behavior between mothers raising CONT and APA litters was obtained. Specifically, no difference in anogenital licking or body licking (Age: *p* = 233, PND: *p* = .567, Age × PND: *p* = .603 and Age: *p* = .756, PND: *p* = .054, Age × PND: *p* = .064, respectively; total licking: Age: *p* = .588, PND: *p* = .220, Age × PND: *p* = .242; Supplementary Fig. 4a, b) was found, and arched-back nursing as well as passive nursing occurred with a similar frequency in CONT and APA mothers (Age: *p* = .478, PND: *p* = .446, Age × PND: *p* = .991 and Age: *p* = .183, PND: *p* < .001, Age × PND: *p* = .559, respectively; total nursing: Age: *p* = .249, PND: *p* < .001, Age × PND: *p* = .755; Supplementary Fig. 4c) was evident. Moreover, the frequency mothers or pups spent outside the nest was not affected by APA (Age: *p* = 250, PND: *p* < .001, Age × PND: *p* = .726 and Age: *p* = .892, PND: *p* = .040, Age × PND: *p* = .915, respectively; Supplementary Fig. 4d, e). Finally, latencies to retrieve the first and the last pup also did not differ between CONT and APA mothers (Age: *p* = .196, PND: *p* < .001, Age × PND: *p* = .906, respectively; Supplementary Fig. 4f). Likewise, no evidence for differences in maternal behavior between mothers raising CONT and APA litters was obtained in the second cohort used for the assessment of hippocampal neuronal plasticity and miRNAs (not shown).

#### Hippocampal neuronal plasticity and microRNAs

**Fig. 6:**
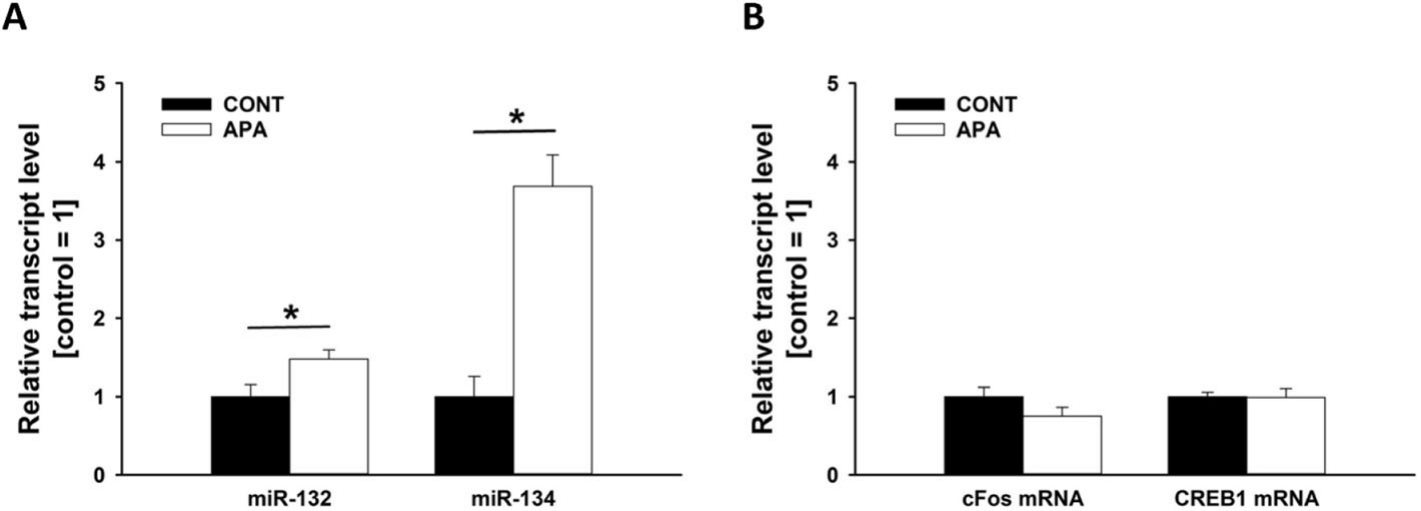
Increased microRNA expression levels in APA rat hippocampal tissue. **a** qRT-PCR analysis of miR-132 and miR-134 from a subset of hippocampi obtained from adult male control (CONT, *n* = 12) and advanced paternal age (APA, *n* = 12) rats. **b** qRT-PCR analysis of cFos mRNA and CREB1 mRNA from a subset of hippocampi obtained from adult male control (CONT, *n* = 12) and advanced paternal age (APA, *n* = 12) rats. Values represent mean transcript levels ± SEM relative to U6 small nuclear RNA (Rnu6). *p < .05

In order to identify potential molecular pathways causally involved in ASD-related behavioral phenotypes seen in APA rats, we studied the expression of miRNAs in the rat hippocampus. The short non-coding miRNAs act as post-transcriptional regulators of gene expression by complementary binding to the 3′ untranslated region of target mRNAs and mediate experience-dependent changes in brain plasticity [52]. Specifically, we measured the expression of the precursor and/or mature miR-132 and miR-134, with miR-132 known to positively regulate dendritic morphogenesis, synaptic plasticity, and neurogenesis [53–56], and miR-134, a negative regulator of dendritic spine size and required, together with other miR-379–410 members, for activity-dependent dendritogenesis in rat hippocampal neurons [57–59]. While pre-miR-132 and pre-miR-134 levels were not affected by APA (*p* = .239 and *p* = .332; respectively), strong effects on the mature miRNAs were evident. Specifically, miR-132 (*p* = .023) and miR-134 (*p* < .001) were both upregulated in APA rats (see Fig. 6a), whereas transcript levels of cFos (*p* = .156) and CREB1 (*p* = .947) were not affected by APA (see Fig. 6b). Of note, all measured transcript levels did not differ between brain hemispheres and no evidence for an interaction between APA and hemisphere was obtained (all *p* values > .05).

## Discussion

Children born to older fathers (typically 40 years and older) have an increased risk of developing neurodevelopmental disorders, such as ASD [4–8]. There is evidence for accumulating risk across generations and a dose-response effect with ASD risk monotonically increasing with paternal age. The neurobiological effects of APA and the pathophysiology underlying increased ASD risk were investigated in our study.

Here, in a large sample of healthy humans, we provided evidence for an association of APA with the personality trait neuroticism, a general risk factor for neuropsychiatric disorders [60], and with schizotypal traits, both related to interpersonal problems. A corresponding phenotype was obtained in our rat model, where we found that APA rats display social-communication deficits emitting fewer pro-social 50-kHz USV than CONT rats. APA rats further displayed repetitive and stereotyped behavioral patterns, together with higher levels of anxiety-related behavior during early development. Importantly, cognitive functioning was intact in APA animals and no evidence for behavioral phenotypes with relevance for depression, nor other neurodevelopmental disorders, such as SZ and BPD, was obtained, highlighting the specificity of the observed behavioral alterations linked to ASD. At the neurobiological level, strong effects of APA on miR-132 and miR-134, implicated in neuronal morphogenesis and dendritic growth, were evident in our rats, with both transcript levels being upregulated in the hippocampus of APA rats. Correspondingly, in humans, the volumes of the right hippocampus and the right IFG as well as the integrity of the fiber bundle connecting these two brain structures, the uncinate fasciculus, were positively correlated with paternal age. Our genome-wide methylation analysis in humans showed suggestive, yet non-significant, differential methylation in APA individuals in *CDH9* and *ZNF266*, linking APA to epigenetic mechanisms which have previously been implicated in synaptic plasticity and neuronal migration. This is the first study demonstrating an association between APA and offspring brain morphology in healthy adult humans. This might be caused in part by alterations in DNA methylation and changes in the expression of miRNAs regulating neuronal plasticity.

### Behavioral phenotypes

#### Personality traits and cognition in humans

In the human sample, effects of APA on personality profiles were detected. These were present in the SPQ-B (all subscales and composite score) in that APA paternal age was correlated with higher levels of schizotypal personality. In addition, an effect on neuroticism was identified. These findings are in line with research on ASD and SZ in which elevated levels of these traits were found patients and more importantly, their relatives [13]. The fact that these effects were detected in healthy individuals underlines the importance of APA-associated effects on the general population. Neuroticism and schizotypy are risk factors for ASD and SZ, independently of APA. Only one other study reported an influence of paternal age on the personality trait “absorption” of the Multidimensional Personality Questionnaire, in line with our findings [61]. We did not find an APA effect on cognitive measures, probably because of ceiling effects in our tests, due to their crudeness and our population of mostly students. Previous studies have demonstrated an inverted u-shaped curve of APA on general intelligence and social skills [62]. Specifically, offspring of fathers younger than 20 years or older than 45 years had a 1.72-fold increased risk to develop an unfavorable set of social skills compared to offspring of fathers with an age between 25 and 29 [63], again in line with our finding of APA correlating with offspring schizotypy.

#### Behavioral phenotypes in rats

In the animal model, we obtained evidence for an ASD-related behavioral phenotype in APA rats, including social-communication deficits, repetitive and stereotyped patterns of behavior, and elevated levels of anxiety-related behavior during early development, together with alterations in developmental milestones. Firstly, during rough-and-tumble play, APA rats displayed social-communication deficits and emitted fewer pro-social 50-kHz USV than CONT rats across all three rough-and-tumble play days. Such pro-social 50-kHz USV are believed to reflect positive affective states (“rat laughter” [64]) and serve important communicative functions as social contact calls, which induce social approach behavior in recipients [65]. The social-communication deficits displayed by APA rats are consistent with findings obtained in mouse studies. Specifically, Smith et al. [17], who used a mouse model to study APA effects on ASD-related behavioral phenotypes in offspring for the first time, observed reduced direct reciprocal social interaction behavior in offspring of old fathers. In a follow-up study, Janecka et al. [66] investigated the impact of APA on developmental trajectories of social behavior in a sex-specific manner and found detrimental effects primarily in adult male mice. Sampino et al. [67] even reported that sociability and social novelty preference can be negatively affected by grand-paternal age in mice. A similar mouse model established by Foldi et al. [68], however, did not display alterations in social behavior, as quantified in T-maze social preference and novelty tasks [69]. Moreover, Ehninger et al. [70] and Milekic et al [21]. did not detect detrimental effects of APA on social behavior in mice using the three-chambered social approach assay. Together, this might suggest that APA does not primarily affect social motivation but mainly social-cognitive functioning most reliably detectable in direct reciprocal social interaction tasks. Here, the present study is the first to demonstrate APA effects on pro-social ultrasonic communication in rats.

Secondly, APA rats displayed repetitive and stereotyped patterns of behavior. Specifically, APA rats showed more circling behavior during tail-chasing than CONT rats, while self-grooming behavior and locomotor activity did not differ between groups. APA effects on repetitive and stereotyped patterns of behavior were also reported by Sampino et al. [67] in mice, with offspring from old fathers displaying more self-grooming behavior than offspring from young fathers. Importantly, in the present study, repetitive and stereotyped patterns of behavior were for the first time not just detected at the motor level but also at a higher cognitive level. While CONT and APA rats displayed intact spatial learning abilities in a radial eight arm maze, APA rats made more perseveration errors during reversal learning.

In addition to social-communication deficits and repetitive and stereotyped patterns of behavior, APA rats displayed higher levels of anxiety-related behavior during early development. Specifically, emission of isolation-induced 40-kHz USV, typically used as an early anxiety measure in the first 2 weeks of life [71], was affected by APA, with USV emission being higher in APA than CONT rats. This is in line with mouse findings obtained by Sampino et al. [67], who also reported increased emission rates in offspring from old fathers, as compared to young fathers. During early development, CONT and APA pups further differed in body weight gain, temperature regulation, and righting reflex, yet this is unlikely to be related to the APA effects on USV emission because these differences were also present at PNDs where USV rates did not significantly differ between CONT and APA rats. In adulthood, however, no evidence for an effect of APA on anxiety-related behavior was obtained in the elevated plus maze. This is in line with most previous findings [17, 21], yet Luo et al. [72] reported decreased anxiety-related behavior in the open field, in rats from old vs. young fathers, while opposite findings were obtained by Sampino et al. [67] in mice. In the elevated plus maze, however, no APA effects on anxiety-related behavior was obtained in previous findings [21, 67, 68, 72] (but see ref. [73]), apart from increased risk assessment in the mouse studies by Foldi et al. [68] and Sampino et al. [67]. Startle responsivity did not differ between CONT and APA rats, consistent with most mouse findings [67, 68], except for Milekic et al. [21] reporting reduced startle responsivity in offspring from old fathers, as compared to young fathers.

Importantly, no evidence for behavioral phenotypes with relevance for depression, nor other neurodevelopmental disorders, such as SZ or BPD, was obtained, highlighting the specificity of the observed behavioral alterations linked to ASD. Specifically, sucrose preference used to assess anhedonia-like alterations with relevance to depression was not affected by APA, consistent with previous findings obtained in mice and rats [67, 72]. Likewise, PPI of acoustic startle was not affected by APA, in line with mouse findings obtained by Sampino et al. [67]. When modeling mania-like elevated drive, i.e., exposing rats to amphetamine, CONT and APA rats displayed an increase in locomotor activity of similar strength in our study. Moreover, the emission of amphetamine-induced 50-kHz USV, possibly reflecting mania-like elevated mood [49, 50], did not differ between our CONT and APA rats. Increases were of similar strength in both experimental groups, indicating that APA effects on 50-kHz USV production specifically occur in a social context, rough-and-tumble play, but not in a non-social one, namely amphetamine treatment.

Finally, no evidence for other cognitive deficits was detected—in line with our human results, such as the novel object recognition paradigm and spatial learning abilities. APA effects on cognitive functioning have rarely been assessed so far. Auroux [74] reported a decrease in learning capacity in offspring with increasing paternal age in rats exposed to avoidance conditioning. Similarly, Luo et al. [72] reported impaired conditioned fear responses and reduced spatial memory in the Morris water maze, yet effects were primarily seen in female but not male rats. Discrepancies might thus be due to multiple factors, including sex and emotional valence.

A controversial issue concerns the pathways through which APA influences rat offspring behavior [26, 37, 75]. First, APA effects could be due to fathers influencing offspring development through direct care of offspring. Because male rats were separated from the females used for breeding before delivery, they were never in contact with their offspring, ruling out this direct paternal influence.

Second, there is growing evidence that, even in the absence of direct contact with the offspring, fathers could achieve an indirect influence on offspring through affecting the quality of mother–infant interactions [26]. Specifically, reduced mate quality associated with APA might result in a shift in maternal investment in offspring growth and development, resulting in either reproductive compensation or differential allocation. The compensation hypothesis suggests that females paired with an unattractive low-quality male will increase their reproductive investment toward offspring to compensate for any disadvantages they may inherit from their father [76]. Alternatively, the differential allocation hypothesis predicts that maternal care behavior is increased in females paired with an attractive high-quality male, particularly if the cost of reproducing is high [77, 78]. Despite conflicting predictions, support for both hypothesis has been provided across a wide variety of species, including mallards [79, 80] and zebra finches, with female zebra finches laying heavier eggs and having offspring with larger growth rates when paired with males that have been artificially made more attractive [81]. In mice, females mated with males that had experienced social enrichment across their lifespan show elevated levels of nursing behavior and anogenital licking toward their offspring [82]. As the direct pathway through contact with the offspring, however, this indirect pathway through altered quality of mother-infant interactions can be ruled out in our study as well, since no evidence for APA effects on maternal behavior was obtained in two independent cohorts of rats despite a very detailed maternal care observation procedure. Specifically, anogenital/body licking and arched-back/passive nursing occurred with a similar frequency in CONT and APA mothers. Moreover, the frequency mothers or pups spent outside the nest was not affected by APA. Finally, latencies to retrieve the first and the last pup did not differ between CONT and APA mothers. This is the first study demonstrating that APA effects are not mediated through changes in maternal investment, a potential confound not addressed so far.

### Mechanisms underlying APA phenotype

#### Brain morphology

Regarding neuroimaging phenotypes in humans, the volume of the right hippocampus and the right IFG were found to be positively correlated with paternal age in our study. These areas have previously been implicated in social cognition in healthy subjects [83, 84]. They are also typically found to be larger in ASD than in controls [85, 86], and are also typically affected in SZ patients [87]. In pediatric [88] as well as adult [89], ASD increased local GM brain volume in the prefrontal cortex and large portions of the left temporal cortex is predictive of greater ASD severity. Better communication skills are associated with greater GM volume in frontal regions (especially the left middle frontal gyrus) and reduced severity of ASD symptoms is associated with greater GM volume in the right IFG [90].

Importantly, the right UF was positively correlated with APA. This white matter bundle connects the right hippocampus and the right IFG. It has been previously implicated in social cognitive functions [91]. Increased FA in frontal tracts has been found in infants with ASD [92]. Studies investigating white matter microstructure in SZ but also in ASD reported mixed results with some studies reporting no differences or lower FA in the UF in SZ [93]. In ASD, the UF is typically found to have lower FA [94, 95], but the opposite effect has also been reported [96].

In summary, the right hippocampus, IFG, and their connection, the UF, have been previously related to social cognitive-emotional functions and show alterations in ASD and SZ patients. APA might particularly impact on these brain areas, which undergo major changes during childhood and adolescence in terms of axonal migration and synaptic plasticity [97].

#### MicroRNA regulation

To elucidate synaptic regulatory mechanisms in APA offspring, we investigated miRNAs in rats. Guided by human brain imaging and previous findings [36, 59, 98, 99], we focused our analyses on hippocampal miRNA expression. We found that two known plasticity-regulating miRNAs, miR-132 [53–56] and miR-134 [57–59], were significantly upregulated in the hippocampus of APA rats, suggesting that miRNA-dependent inhibition of synaptic genes could play a role in the development of ASD-related behavioral phenotypes associated with APA. In fact, both miRNAs are associated with social behavior and ultrasonic communication in rats [59, 98, 99]. For instance, post-weaning social isolation leads to increased hippocampal miR-134 expression [59] and this increase is associated with social communication deficits [98]. Most recently, we have shown that members of the miR379-410 cluster, including miR-134, inhibit sociability in mice [36]. Intriguingly, known target genes of miR-132 and miR-134, MeCP2 and Limk1, are mutated in the neurodevelopmental disorders Rett syndrome and Williams syndrome, respectively [100, 101]. Both syndromes present, among other symptoms, social abnormalities and repetitive behaviors. It is therefore tempting to speculate that impaired expression of MeCP2 and Limk1 downstream of miRNAs could contribute to ASD-related behavior of APA rats reported here. Future experiments are needed to fully reveal and characterize miRNA-target interactions in the APA rat model and their significance for the development of ASD-related behavioral phenotypes. Moreover, in light of the repetitive and stereotyped behavioral patterns displayed by APA rats, analyses of striatal miRNA expression levels appear warranted. In the human sample, surprisingly, we detected a significant downregulation of miR-134 levels in peripheral blood of the offspring of older compared to younger fathers, which contrasts to our findings from the brain of APA rats. Intriguingly, a similar dichotomy between miRNA expression in brain vs. blood was recently reported for SZ. In SZ patients, miR-134 was found to be upregulated in the dorsolateral prefrontal cortex [102] while being downregulated in peripheral blood [103]. The mechanistic underpinnings of this opposite regulations are currently unknown and clearly deserve future investigation. Interestingly, miR-134 is encoded by an imprinted gene, bearing important implications: It is widely held that DNA methylation differences occurring during spermatogenesis are not transmitted to offspring due to the reprogramming of methylation pattern that occurs in the embryo after fertilization. Generally, reprogramming in the preimplantation embryo results in demethylation of the paternal genome and subsequent remethylation according to the maternal genome [104]. Imprinted genes, however, escape reprogramming and the paternal methylation pattern is inherited by the offspring [105] and thus could transfer paternal effects on offspring behavior.

#### Epigenetic effects

One step further down a potential causal pathway is epigenetic regulation of brain development [26]. In humans, our genome-wide methylation analysis of paternal age did not reveal significantly differentially methylated CpG sites after correction for multiple testing. This may reflect at least in part that the sample size of the present study was too small to detect differentially methylated CpGs at a genome-wide level. Three CpGs, however, showed a nominal *p* value < 1 × 10^−6^ implicating *CDH9* and *ZNF266*. *CDH9* encodes for cadherin 9, which is a member of the cadherin superfamily of calcium-dependent cell-cell adhesion molecules [44]. Cadherins play an important role in the regulation of neuronal migration, gray matter differentiation, neural circuit formation, spine morphology, and synapse formation [106]. Notably, CDH9 is highly expressed in the human brain, particularly in the frontal cortex (http://www.gtexportal.org/home/) [107] and has previously been reported as a genome-wide significant risk gene for ASD [108]. *ZNF266* encodes for the zinc finger protein 266. The biological functions of zinc finger proteins are highly diverse and include DNA recognition and transcriptional regulation [109]. Interestingly, zinc finger proteins have been implicated in the development of neuropsychiatric disorders, e.g., *ZNF804a* in SZ and BPD [110, 111].

## Limitations

There are some limitations to the present study: First, even though the ASD-like behavior observed in rats could not directly be observed in humans, it was paralleled in the SPQ-B questionnaire. Second, results of brain morphology in humans were not corrected for multiple testing, but given that DTI results strongly support VBM findings lend support to reported findings. Third, we did not carry out epigenetic analyses in rats, thus this limits the generalizability of the results.

## Conclusion

In the present study, effects of APA emerged on fronto-hippocampal brain structures and personality profiles (neuroticism, schizotypy) in the human cohort. In the rat model, a phenotype consistent with the results detected in humans was found, namely social-communication deficits, repetitive and stereotyped patterns of behavior, and elevated levels of anxiety-related behavior during early development, together with alterations in early development. Potential underlying mechanisms may be rooted in differential DNA methylation and miRNA regulation. It is noteworthy that the effects observed in humans were found in a sample of healthy subjects. This is especially important as it could be hypothesized that effects might have even been stronger in a sample of patients with ASD or SZ, thus testing the effects of APA in healthy subjects could be considered a more conservative test of the effects of APA than in patients. This is the first study demonstrating an association between APA and offspring brain morphology and connectivity in healthy adult humans, which might be due to alterations in DNA methylation and changes in the expression of miRNAs regulating neuronal plasticity.

## Supplementary information

**Additional file 1.** Supplementary Material.
