# Supplementary material for: Advanced paternal age as a risk factor for neurodevelopmental disorders: a translational study
Source: Mol Autism. 2020 Jun 23;11:54. doi: 10.1186/s13229-020-00345-2 (PMC7310295; doi:10.1186/s13229-020-00345-2)
Supplement: Supplementary file 1 — Additional file 1. Supplementary Material. [file 13229_2020_345_MOESM1_ESM.docx]

**Supplementary Material**

1. **Human Sample**
   1. **Behavioral data acquisition and analyses**

All subjects were tested with the NEO-FFI[1] and the SPQ-B.[2] In addition, subjects underwent neuropsychological testing including the d2-attention test,[3] brief verbal intelligence testing (Mehrfach-Wortwahl-Test Version B; MWT-B),[4] Trail-making test,[5] verbal fluency (Regensburger Wortflüssigkeits Test, RWT),[6] letter-number-sequencing,[7] number-symbol coding (WAIS-IV),[8] spatial-span (WMS III),[9] and the verbal learning and memory test (VLMT).[10] Statistical analyses were carried out with SPSS 15.0 (SPSS Inc., 2006). For the behavioral data, partial correlation analyses were calculated for paternal age, all SPQ-B scores (including the sum score) and the NEO-FFI subscales. Maternal age, subjects’ age, education, and sex were entered as covariates.

- 1. **Structural MR image acquisition in humans**

For sixty-nine data sets, imaging was performed on a 3-Tesla Trio MR scanner (Siemens Medical Systems) in the Institute of Neuroscience and Biophysics – Medicine, Research Center Jülich, Germany. Acquisition matrix was 256*256, 176 slices, thickness 1 mm, acquisition parameters were as follows: TE 3.03, TR 2250, flip angle 9°, distance factor 50 per cent, field of view 256 mm, resulting in isotropic voxels of 1x1x1 mm3 size. For the remaining data sets (N=273), imaging was performed on a 3-Tesla Trio MR scanner (Siemens Medical Systems) at the Department of Psychiatry and Psychotherapy, Philipps-University Marburg, Germany. Acquisition matrix was 256*256, 176 slices, thickness 1mm, acquisition parameters were TE 2.26, TR 1900, flip angle 9°, distance factor 50 per cent, field of view 256 mm, resulting in isotropic voxels of 1x1x1 mm^3^ size. For both scanners, head movements were minimized by tightly fixating the head with cushions during the scanning procedure. After a localizer sequence, a high-resolution T1-weighted volume data set of the whole brain was acquired using an MP-RAGE (Magnetization Prepared Rapid Gradient Echo) sequence. Slices covered the whole brain and were positioned transaxially parallel to the anterior-posterior commissural line (AC-PC).

- 1. **DTI MRI image acquisition**

Two-hundred-twenty-two DTI data sets of the Marburg sample (see above) were available and acquired as described in the following: Diffusion weighted images (DWI) were acquired with a single-shot echo-planar imaging (EPI) sequence (repetition time 7800 ms, echo time 90 ms, 2.5 mm isotropic resolution, 56 slices in interleaved order, no gap, 30 gradient directions, b-values = 1000 s/mm^2^, parallel imaging (GRAPPA) with factor 2, bandwidth 1502 Hz/pixel, phase encoding direction AC-PC). For each participant two subsequent sets of DWI and five interleaved non-weighted images (b-values 0) were acquired.

- 1. **Structural image pre-processing**

All data sets were pre-processed with standard (=default) parameters of the VBM8-Toolbox (http://dbm.neuro.uni-jena.de/vbm/) in SPM8. Images were inspected for segmentation and normalization and sample homogeneity was controlled using covariance statistics. All volumes outside 2SD of the mean were removed from further analyses. In a final step, an 8 mm Gaussian filter was applied to images with the aim to obtain smoothed modulated normalized data representing grey matter volume for the statistical analysis.

- 1. **DTI pre-processing**

Analysis of DTI data was performed using the FMRIB Software Library (FSL version 5.02, Oxford, U.K., http://www.fmrib.ox.ac.uk/fsl). DTI pre-processing was performed as follows: (i) head motion and eddy current correction, (ii) removal of non-brain tissue from an image of the whole head, and (iii) fitting of diffusion tensor model at each voxel. All FA images were visually checked for artifacts, intensity range problems, and general data quality.

- 1. **VBM second-level analysis**

For the combined sample of N=342 subjects, a multiple regression analysis was calculated in SPM8. Paternal age, maternal age, subject’s age, sex, scanning site and level of education were entered in the model. To restrict analyses to areas with a grey matter probability of .2 images were thresholded with an absolute threshold of .2. Contrasts were calculated for the positive and negative effect of paternal age and results were thresholded at .001 uncorrected and restricted to cluster > 50 voxels.

- 1. **DTI second-level analysis**

Voxelwise statistical analysis of the fractional anisotropy (FA) maps of N=222 subjects was performed using Tract-Based Spatial Statistics (TBSS) version 1.2 implemented in FSL according to the standard procedure[11]. A multiple regression analysis was performed within the framework of a general linear model (GLM) using the FSL Randomise tool. Paternal age, maternal age, subject’s age, sex, and level of education were entered in the model. Each contrast (positive and negative effect of paternal age) was performed according to permutation-based non-parametric inference with 10,000 random permutations and a significance level of P<0.05 family-wise error (FWE) corrected using threshold-free cluster enhancement (TFCE).[12]

- 1. **Genome-wide methylation analysis**

Lymphocyte DNA was isolated from venous blood samples using standard methods. Bisulfite conversion of the DNA samples was performed using the EpiTect Bisulfite Kit (Qiagen, Hilden, Germany). The bisulfite-treated DNA was analyzed using the Infinium HumanMethylation450 BeadChip which covers over 450,000 cytosine-phosphate-guanosine dinucleotide (CpG) sites per sample (Illumina, San Diego, CA, USA). The processing of the arrays was performed in accordance with the manufacturer’s protocol using an automated pipeline, and the arrays were scanned on an Illumina iScan platform (Illumina, San Diego, CA, USA). We used the lllumina GenomeStudio Methylation Module (v1.8, Illumina, San Diego, CA) to extract raw DNA methylation signals without background normalization.

We processed the raw image data (.idat files) using the GenomeStudio Methylation Module (v1.8, Illumina, San Diego, CA) and applied the R/Bioconductor packages minfi[13] and limma[14] for downstream and statistical analyses. We performed state-of-the-art data processing steps including quality control, bead filtering, preprocessing of the raw data (background subtraction and color-bias adjustment), normalization, batch effect correction and correction for blood cell type heterogeneity. We confirmed the reported sex by assessing the methylation status on the X and Y chromosome. Probes with a detection p-value >0.05 and a beadcount <3 in >20% of samples were excluded. For all samples, β-values were calculated from the intensity of the methylated (M) and unmethylated (U) alleles ratio of fluorescent signals. For downstream analysis β-values were transformed into M-values to increase the performance in terms of the detection and true positive rates for highly methylated and unmethylated CpG sites.[15] We visually inspected multidimensional scaling (MDS) plots to evaluate the effects of known confounding variables. For batch effect correction we applied ComBat[16] from the R/Bioconductor package sva[17] . Analysis of variance (ANOVA) was conducted to identify the contribution of technical (amplification plate, slide, array) and biological (sex, age, medication) variables before and after batch effect correction. We estimated blood cell type proportions using the regression calibration approach algorithm.[18] Subsequently, we included the cell type proportion estimates as covariates in the linear regression models. For the genome-wide methylation analysis of paternal age as a categorical trait, we applied linear regression models on M-values using the R/Bioconductor package limma and adjusted the model for the subject’s age, the maternal age, the SPQ-B score, the disorganization score and the cell type proportion estimates. We applied the Benjamini-Hochberg method to correct for multiple testing.

# 2.9 Gene expression measurement in the human cohort

# Whole blood was collected in a PAXgene Blood RNA Tube and stored until analysis at -80°C[19]. Total RNA including miRNA was then extracted using PAXgene Blood miRNA Kit (Qiagen), following the manufacturer´s protocol. The concentration of the RNA samples was measured with the Qubit RNA BR Assay Kit (Life Technologies) and the sample purity was assessed with NanoDrop 1000 Spectralphotometer (Thermo Scientific). Afterwards, the cDNA was then synthesized by a reversed transcription reaction using the TaqMan MicroRNA Reverse Transcription Kit (Applied Biosystems) with the RT primer from the TaqMan MicroRNA Assays mmu-miR-134 (Assay ID 001186), hsa-miR-132 (Assay ID 000457) and U6 snRNA (Assay ID 001973) (all by Applied Biosystem) with following the protocol for single-stranded small RNA. Quantitative PCR was performed on the QuantStudio 7 Flex Real-Time PCR System (Applied Biosystems by Life Technologies) by using TaqMan Universal PCR Master Mix, no AmpErase UNG (Applied Biosystems), and the TaqMan MicroRNA Assays mmu-miR-134 and hsa-miR-132. TaqMan MicroRNA Assay U6 snRNA was used as an internal standard. All results were calculated with the QuantStudio Real-Time PCR Software v1.3 (Applied Biosystems by Thermo Fisher Scientific) by the comparative 2^−ΔΔCt^ method and normalized to U6 snRNA. Analyses were carried out in triplicate.

1. **Animals and Housing**

Male and female Wistar rats (RccHan:WIST; Harlan, Venray, The Netherlands) served as subjects and were housed in an animal room with a 12:12 h light/dark cycle (lights on 8-20 h) where the environmental temperature was maintained between 20-25° Celsius (humidity: 30-50 %). Rats were kept in Macrolon type IV cages (380 x 200 x 590 mm, plus high stainless steel covers; Tecniplast Deutschland GmbH, Hohenpeißenberg, Germany) on Tapvei peeled aspen bedding (Indulab AG, Gams, Switzerland), with lab chow (Altromin, Lage, Germany) and water (0.0004% HCl-solution) available ad libitum unless specified otherwise (for details, please see below). For identification, pups were marked with a paw tattoo using non-toxic animal tattoo ink after testing on postnatal day 5 (PND 5; Ketchum permanent Tattoo Inks green paste, Ketchum Manufacturing Inc., Brockville, ON, Canada). The ink was inserted subcutaneously through a 30-gauge hypodermic needle tip into the center of the paw. All experimental procedures were performed according to the legal requirements of Germany and approved by the ethical committee of the local government (MR 20/35 Nr. 33/2012; Regierungspräsidium Gießen, Germany). Two cohorts of rats were tested.

- 1. **Experimental manipulation**

**First cohort – comprehensive longitudinal behavioral phenotyping:** Female rats aged 2 months were bred with young male rats of 2 months (N=10 females; control – CONT) or old male rats of 12 months (N=10 females; advanced paternal age – APA). For breeding, 1 male rat (CONT: N=5; APA: N=5) was housed with 2 female rats for 2 weeks. Breeding resulted in N=16 litters, namely N=9 CONT and N=7 APA litters. Total progeny generated was N=99 pups (female: 49; male: 50) under CONT conditions and N=81 pups (female: 37; male: 44) under APA conditions (no APA effect on sex ratio was observed; p=.610). Average litter size for CONT litters was 11.00±1.32 pups (females: 5.44±0.77; males: 5.56±1.13) and for APA litters 11.57±0.84 pups (females: 5.29±0.87; males: 6.29±0.87). At day of birth (PND 0), litters were culled to N=8 pups per litter, with N=4 females and N=4 males whenever possible, to avoid litter size effects on maternal care behavior and comprehensive longitudinal behavioral phenotyping.[20] Moreover, 2 CONT litters were excluded completely from comprehensive longitudinal behavioral phenotyping to obtain N=7 litters per experimental conditions. Behavioral assays conducted after weaning were performed in male offspring due to higher ASD prevalence in males than females in humans. In the APA condition, N=2 male rats from different fathers had to be sacrificed due to teeth anomalies. Behavioral assays were thus performed with N=28 CONT male rats and N=25 male APA rats.

**Second cohort – hippocampal neuronal plasticity and miRNAs:** Female rats aged 2 months were bred with young male rats of 2 months (N=10 females; control – CONT) or old male rats of 21 months (N=10 females; advanced paternal age – APA). For breeding, 1 male rat (CONT: N=5; APA: N=5) was housed with 2 female rats for 2 weeks. Breeding resulted in N=17 litters, namely N=9 CONT and N=8 APA litters. Total progeny generated was N=106 pups (female: 55; male: 51) under CONT conditions and N=85 pups (female: 38; male: 47) under APA conditions (no APA effect on sex ratio was observed; p=.324). Average litter size for CONT litters was 10.60±1.36 pups (females: 5.50±0.70; males: 5.10±0.82) and for APA litters 9.44±1.20 pups (females: 4.22±0.79; males: 5.22±0.86). At day of birth (PND 0), litters were again culled to N=8 pups per litter, with N=4 females and N=4 males whenever possible. All hippocampal neuronal plasticity measurements were performed in male offspring only. Two males per litter were included in the analysis (out of the first 12 litters; 6 CONT, 6 APA). Measurements were thus performed with N=12 CONT male rats and N=12 male APA rats.

- 1. **Comprehensive longitudinal behavioral phenotyping**
     1. **Experimental Overview**

**First cohort – comprehensive longitudinal behavioral phenotyping:** Rats were exposed to a comprehensive set of behavioral phenotyping assays, including well-established paradigms with high relevance and sensitivity for the diagnostic and associated symptoms of ASD[21] and other neuropsychiatric disorders, such as anxiety and depression, but also SZ and BPD.[22–24] A longitudinal design was applied. In the first two weeks of life, maternal care behavior (PND 2, 4, 6, 8, 10, and 12) was observed and isolation-induced 40-kHz ultrasonic vocalizations (USV) were assessed together with developmental milestones (PND 5, 7, 9, and 11). Following weaning at PND 21, rats were housed in same-sex groups of N=3-5. Shortly after weaning, playback of pro-social 50-kHz USV (PND 26±1) was performed and rough-and-tumble play behavior and pro-social 50-kHz USV (PND 32-34) were measured, with findings of the former paradigm being reported elsewhere. Then, after 3 days of standardized handling for 5 min (PND 61-63±3), repetitive and stereotyped patterns of behavior (PND 64±3) were assessed, followed by open field (PND 68-69±3), elevated plus maze (PND 73±3), and novel object recognition (PND 78-79±3). Thereafter, half of the rats were trained in the spatial learning and reversal learning tasks (PND 114-123±4), while the other half was exposed to the sucrose preference test (PND 114-123±4). This was followed by the assessment of acoustic startle and pre-pulse inhibition (PPI) of acoustic startle (PND 188±6), and amphetamine-induced hyperactivity and positive 50-kHz USV (PND 194-196±6). All behavioral assays were conducted during the light phase of the light/dark cycle between 8-20 h. Prior to each test, behavioral equipment was cleaned using a 0.1 % acetic acid solution followed by drying.

**Second cohort – hippocampal neuronal plasticity and miRNAs:** In the first two weeks of life, maternal care behavior (PND 2, 4, 6, 8, 10, and 12) was observed. Following weaning at PND 21, rats were housed in same-sex groups of N=2-5. After 3 days of standardized handling for 5 min (PND 88-90±5), novel object recognition (PND 91-92±5) was performed. Thereafter, acoustic startle and PPI of acoustic startle (PND 200±5) and amphetamine-induced hyperactivity and positive 50-kHz USV (PND 232-234±5) were determined. Finally, PPI under different pharmacological conditions was conducted, namely PPI following saline injection (PND 263±5), PPI following 0.5 mg/kg apomorphine injection (PND 267±5), and PPI following 1.0 mg/kg apomorphine injection (PND 271±5). Findings of the latter three behavioral paradigms are reported elsewhere. Brains were taken out at PND 312±5.

- - 1. **Maternal care**

On PND 2, 4, 6, 8, 10, and 12, maternal care behavior was observed, using a modified protocol previously established.[25] Each litter was housed with its dam on Tapvei peeled aspen bedding (Indulab AG) in a Macrolon type IV cage (380 x 200 x 590 mm, plus high stainless steel covers; Tecniplast Deutschland GmbH) that permitted a clear view from all sides. Continuous observations were made at regular time points each day. Specifically, four observations lasting 1 h each were made during the light phase of the light/dark cycle (9-10 h, 11-12 h, 14-15, and 16-17 h). These observation periods were selected since maternal behavior occurs more frequently during the light phase.[26] Within each observation period, the behavior of each mother was scored every minute (60 observations/period x 4 observation periods per day = N=240 observations/mother/day, resulting in N=1.440 observations per mother over the 6 days; total observation duration of 24 h). The occurrence of the following behaviors was scored: anogenital licking, body licking, arched-back nursing, passive nursing, mother outside nest, and pup outside nest. Dams were not disturbed during maternal care observations. In addition, retrieval behavior was measured between 12-13 h. First, the mother was removed from the housing cage and isolated in a Makrolon type III cage (265 x 150 x 425 mm, plus high stainless steel covers; Tecniplast Deutschland GmbH). Then, pups were scattered over the floor of the housing cage and the mother was reintroduced. Latencies to retrieve the first and the last pup were measured. Observations ended when all pups were retrieved or when 5 min had elapsed.

- - 1. **Isolation-induced 40-kHz ultrasonic vocalizations and developmental milestones**

On PND 5, 7, 9, and 11, pups were isolated from their mother and littermates for 5 min under room temperature (20-23 °C) to induce isolation-induced 40-kHz USV, as described previously.[25] Pups were removed individually from the nest at random and gently placed into an isolation box (23 x 28 x 18 cm) made of white and transparent plastic walls. The roof and one wall were made of transparent plastic to allow video observation during the test. The isolation box was placed in a sound attenuating isolation cubicle (51 x 71 x 51 cm; Coulbourn Instruments, Allentown, PA, USA) equipped with 2 white-light LED spots (63 lux, Conrad Electronic GmbH, Hirschau, Germany) and a black/white CCD camera (Conrad Electronic GmbH) connected to an external multimedia hard drive (ScreenPlay Pro HD, Iomega, San Diego, CA, USA). Isolation-induced 40-kHz USV were recorded using an UltraSoundGate Condenser Microphone (CM16; Avisoft Bioacoustics, Berlin, Germany) placed in the roof of the box, 12 cm above the floor. After the 5 min isolation period, body temperature and weight were determined. For body temperature determination, a Testo 110 thermometer with surface sensor (Testo AG, Lenzkirch, Germany) was used. Body weight was measured using a palmscale (PS6-250; MyWeigh Europe, Hückelhoven, Germany). Finally, the righting reflex was assessed by placing pups on their back on a flat, hard surface. A stopwatch was used to determine the time that it took them to right themselves on all four paws (maximum: 30 seconds).

- - 1. **Rough-and-tumble play behavior and pro-social 50-kHz ultrasonic vocalizations**

On PND 32-34, rough-and-tumble play behavior and the emission of pro-social 50-kHz USV were measured, using a modified protocol previously established.[27] On three consecutive days, pairs of juvenile rats were allowed to socially interact for 5 min in an, at first, unfamiliar observation arena (35 x 35 cm, with Plexiglas walls; floor covered with 1 cm of fresh bedding) after one rat of the pair being habituated to the test environment for 2 min. Rats were always paired with a littermate partner from the same experimental condition, since it is not yet possible to identify the sender of pro-social 50-kHz USV during rough-and-tumble play behavior in a reliable manner due to technical limitations. To enhance the level of social motivation, juvenile rats were socially isolated for 24 h prior testing in a Makrolon type III cage (265 x 150 x 425 mm, plus high stainless steel covers; Tecniplast Deutschland GmbH), and isolation was maintained throughout the three days testing period. For behavioral analyses, a digital camera (TK-1281 Color Video Camera, JVC, Yokohama, Japan) was used and connected to an external multimedia hard drive (ScreenPlay Pro HD, Iomega). The following behavioral measures were scored by an experienced observer blind to experimental condition using The Observer XT (Noldus, Wagenigen, The Netherlands): duration of rough-and-tumble play (including pinning, wrestling, and chasing), duration of social investigation (including sniffing the anogenital and head/neck regions of the partner), and duration of physical contact, with the latter two being considered as social but non-playful behaviors. Pinning was defined as one rat lying with its dorsal surface on the floor with the other rat standing over it. Wrestling was scored when a group of play-specific behaviors, including wrestling, boxing, and pouncing, occurred. Chasing was defined as moving in the direction of or pursuing the partner while the partner is moving away. Pro-social 50-kHz USV were recorded using an UltraSoundGate Condenser Microphone (CM16; Avisoft Bioacoustics) placed 35 cm above the floor of the center of the observation arena. Rough-and-tumble play behavior and the emission of pro-social 50-kHz USV were measured under red light (28 lux) conditions.

- - 1. **Repetitive and stereotyped patterns of behavior**

On PND 64±3, repetitive and stereotyped patterns of behavior were tested in a Makrolon type III cage (265 x 150 x 425 mm, plus high stainless steel covers; Tecniplast Deutschland GmbH) without bedding material. For behavioral analyses, a digital camera (TK-1281 Color Video Camera, JVC) was used and connected to an external multimedia hard drive (ScreenPlay Pro HD, Iomega). Behavioral analysis was performed by an experienced observer blind to experimental condition. Repetitive and stereotyped patterns of behavior were assessed by measuring the duration of self-grooming and circling behavior during tail-chasing. For assessing locomotor activity, the test cage was virtually divided in two halves by a line and the number of line crossings and rearing behavior was counted. Repetitive and stereotyped patterns of behavior were tested once under white light (30 lux) conditions for 20 min.

- - 1. **Open field**

On PND 68-69±3, exploratory behavior in a small open field was assessed, as described previously.[25] The open field was made of gray plastic (40 x 40 x 40 cm). Testing began by placing the rat into a corner of the open field, facing a wall. Open field behavior was automatically monitored by means of two grids of infrared sensor beams mounted horizontally 2.5 cm and 14.5 cm above the floor for assessing distance travelled and rearing behavior, respectively (Tru Scan^TM^, Photobeam Sensor-E63-22, Coulbourn Instruments, Allentwon, PA, USA). The time spent in the center was also determined. Open field behavior was tested under red light (28 lux) conditions for 10 min on two consecutive days.

- - 1. **Elevated plus maze**

On PND 73±3, anxiety-related behavior in the elevated plus maze was determined, as described previously.[25] The elevated plus maze was made of gray plastic. It consisted of two opposed open arms and two opposed closed arms (arm sizes: 50 x 10 cm) extending from an open central square (10 x 10 cm). The maze was elevated 50 cm above the floor. Testing began by placing the rat in the center of the elevated plus maze, facing an open arm. For behavioral analyses, a digital camera (EQ150, EverFocus, Taipei, Taiwan) was mounted 1.5 m above the floor of the elevated plus maze and connected to a video recorder (DMR-ES35V, Panasonic, Osaka, Japan). Behavioral analysis was performed by an experienced observer blind to experimental condition. The following behavioral measures were analyzed: number of entries into open or closed arms, time spent on open or closed arms, and the duration of risk assessment. An entry was scored when all four paws crossed into the arm. Risk assessment was scored when the animal’s body was in a stretched position between an open and a closed arm[28]. Elevated plus maze behavior was tested once under white light (30 lux) conditions for 5 min.

- - 1. **Novel object recognition**

On PND 78-79±3, the novel object recognition test was conducted in a large open field, as described previously.[29,30] The open field was made of gray plastic (60 x 60 x 60 cm). Rats were first habituated to the open field (no objects present) by placing them into the box for 20 min. Then, 24 h after the habituation session, the novel object recognition test was conducted, which consisted of three phases: acquisition trial, inter-trial interval and recognition trial. In the acquisition trial, each rat was allowed to freely explore the open field containing two identical sample objects for 5 min. The objects were placed in one of the back corners of the box, 15 cm away from the walls. As objects, either two silver iron cylinders (5 cm in diameter, 8 cm high) or two red metal cubes (5 x 5 x 8 cm) were used in a counter-balanced manner. After the acquisition trial, the rats were returned to their home cages for 30 min, the inter-trial interval. During that time, one clean familiar object and one clean novel object were placed in the open field, where the two identical objects had been located during in the acquisition trial. After the inter-trial interval, each rat was returned to the open field for a 5 min recognition trial and allowed to freely explore the familiar and the novel object. For behavioral analyses, a digital camera (EQ150, EverFocus, Taipei, Taiwan) was mounted 1.5 m above the floor of the open field and connected to a personal computer with Viewer^2^ (Biobserve GmbH, St. Augustin, Germany) for recording and data storage. Object investigation was defined as time spent sniffing the object when the nose was oriented toward the object and the nose-object distance was 5 cm or less. Recognition memory was defined as spending significantly more time sniffing the novel object than the familiar object. The memory index was calculated as follows: (Exploration time novel object) / (Exploration time novel object + Exploration time familiar object). Testing was performed under white light (5 lux) conditions.

- - 1. **Spatial learning and reversal learning**

On PND 114-123±4, half of the rats were trained in the spatial learning and reversal learning tasks, using a modified protocol previously established.[31] Spatial learning and reversal learning was performed on a radial eight arm maze made of black plastic. The arms (9.8 x 40.5 cm) extend radially from a central platform (diameter: 24 cm) and were numbered in a clock-wise fashion from 1 to 8. Each arm had a single transparent plastic wall (20 x 17 cm) affixed to its right side to restrict rats from traversing to adjacent arms without entering the central platform. Four cm from the distal end of each arm, a food pit (5.3 cm in diameter; 4.0 cm deep) was embedded into its floor. The maze was positioned 52 cm above the floor in a testing room with several extra-maze cues. To eliminate distinct odor cues from the baited arms, four containers with food pellets were placed on the floor beneath the center of the maze. To enhance their level of motivation for food, rats were food deprived, with food being withdrawn from home cages except for 1 h of daily free access. For food deprivation, rats were socially isolated in a Makrolon type III cage (265 x 150 x 425 mm, plus high stainless steel covers; Tecniplast Deutschland GmbH). Food deprivation and social isolation started seven days before the beginning of the spatial learning and reversal learning tasks. Starting with the day of radial maze training, the animals received their daily 1 h free access to food in their home cage no earlier than 1h after spatial learning and reversal learning. The spatial learning period lasted six days, immediately followed by the reversal learning period, which lasted four days. Initially, the rats were exposed to the food pellets later used as reward (45 mg, BioServ Dustless Precision Pellets, Flemington, NJ, USA) in the home cage and were habituated to the radial eight arm maze, which then contained food pellets in all arms, both on the arms and in the food pits. A given animal remained in the maze until it had eaten all pellets, or until a cut-off criterion of 30 min was reached. During the six spatial learning days, the rats were tested during four trials per day. For all animals, and during all trials of each spatial learning day, arms 1, 3, 5, 6, and 8 never contained food, whereas arm 2 was consistently baited with six food pellets and arms 4 and 7 were consistently baited with one pellet. On day six of the spatial learning period, the last trial was a probe trial with no food pellets available lasting 5 min. During the four reversal learning days, the rats were again tested during four trials per day. Importantly, however, for all animals, and during all trials of each reversal learning day, arms 1, 2, 4, 6, and 7 never contained food, whereas arm 5 was consistently baited with six pellets and arms 3 and 8 were consistently baited with one food pellet. On day four of the reversal learning period, the last trial was a probe trial with no food pellets available lasting 5 min. For both spatial learning and reversal learning, the maze was cleaned with 0.1% acetic acid and dried thoroughly before each trial. Between trials, the animal was placed into an empty cage next to the radial maze; inter-trial time was 3 min. A trial was ended, if the animals had eaten all food pellets, or if a cut-off criterion of 5 min was reached, except for probe trials. Start positions were rotated, with rats starting from new positions in each trial. Behavior was monitored by a video camera (Panasonic WV- BP 330/GE, Hamburg, Germany) from about 150 cm above the radial maze, which fed into an external multimedia hard drive (ScreenPlay Pro HD, Iomega). For behavioral analysis, an experienced observer blind to experimental condition scored the videos for the type of arm entries (counted if all four paws were placed on that arm) and the time until a trial was completed. Arm entries were scored as either number of baited arms visited, errors of working memory (re-entries into baited arms), errors of reference memory (entries into non-baited arms), “mixed” errors (re-entries into non-baited arms) for both spatial learning and reversal learning periods. In addition, for the first reversal learning day, arm entries were scored either as number of previously baited arms, i.e. arms baited during the spatial learning period, currently baited arms, i.e. arms baited during the reversal learning period, and never baited arms. Spatial learning and reversal learning was tested under dim red light (10 lux) conditions.

- - 1. **Sucrose preference**

On PND 114-123±4, the other half of the rats was exposed to the sucrose preference test. For the sucrose preference test, rats were socially isolated in a Makrolon type III cage (265 x 150 x 425 mm, plus high stainless steel covers; Tecniplast Deutschland GmbH). As the other rats in the spatial learning and reversal learning tasks, rats were food deprived, with food being withdrawn from home cages except for 1 h of daily free access. Food deprivation and social isolation started seven days before the beginning of the sucrose preference test. Initially, rats were trained to drink a 1 % sucrose solution by forced exposure to sucrose in place of water for 24 h. Each animal was then presented simultaneously with 2 bottles, one containing sucrose (1 %), the other one water, for six days. Following these six days, each animal was presented simultaneously with 2 bottles, one containing sucrose (0.5 %), the other one water, for four days. The position of the 2 bottles (right/left) was varied randomly from day to day. During the test, both bottles were removed every 24 h for weighing and replaced by a second pair of pre-weighed bottles.

- - 1. **Acoustic startle and pre-pulse inhibition of acoustic startle**

On PND 188±6, acoustic startle and PPI of acoustic startle were tested using four identical test chambers of the TSE Startle Response System^TM^ (Bad Homburg, Germany), following a previously established protocol.[32] Rats were placed into wire-mesh cages (27 x 9 x 10 cm) on piezo-accelerometers in sound attenuated chambers with two loudspeakers mounted at a distance of 4 cm from the wire-mesh cages. After 5 min of acclimation, five startle stimuli (20 ms white noise pulse of 105 dB sound pressure level (SPL)) were presented in order to habituate the rats to the startle stimuli to obtain a stable baseline. For the assessment of PPI of acoustic startle, rats were exposed to seven different trial types (10 trials each): (1) no stimulus, (2) pulse alone (20 ms white noise pulse of 105 dB SPL), (3) loudest pre-pulse alone (20 ms white noise pulse of 76 dB SPL), and pre-pulse pulse trials, with a pre-pulse of (4) 64 dB SPL, (5) 68 dB SPL, (6) 72 dB SPL, and (7) 76 db SPL, followed by a 105 dB pulse with the onset-onset interval between pre-pulse and pulse set at 120 ms. After assessing PPI of acoustic startle, again five startle stimuli (20 ms white noise pulse of 105 dB SPL) were presented as during the acclimation period. Trials were presented in random order. An inter-trial interval of 20-30 s was used. One test session lasted about 40 min. Background white noise level was set at 60 dB SPL. Acoustic startle response magnitudes were averaged and expressed as arbitrary units. PPI was calculated as percentage of acoustic startle response magnitudes during pulse alone trials, i.e. [100 x (mean ASR amplitude on pulse alone trials – mean ASR amplitude on prepulse-pulse trials)/mean ASR amplitude on pulse alone trials]. One day prior testing, all rats were again handled for 5 min in a standardized way and habituated to the wire-mesh cages.

- - 1. **Amphetamine-induced hyperactivity and positive 50-kHz ultrasonic vocalizations**

On PND 194-196±6, amphetamine-induced hyperactivity and positive 50-kHz USV were assessed, using a modified protocol previously established.[33,34] For testing, the abovementioned small open field (40 x 40 x 40 cm) was used, except that the floor was covered with 1 cm of fresh bedding.[35] Testing occurred on three consecutive days for 45 min each. On day 1, rats were habituated to the open field. On day 2, rats received vehicle (0.9% NaCl; i.p.). On day 3, rats received AMPH (2.5 mg/kg, i.p., Sigma, St. Louis, MO, USA). For USV detection, an UltraSoundGate Condenser Microphone (CM16; Avisoft Bioacoustics) was mounted centrally at 50 cm above the floor of the open field. The recording session started immediately after injection. Amphetamine-induced hyperactivity was automatically monitored by means of two grids of infrared sensor beams mounted horizontally 2.5 cm and 14.5 cm above the floor for assessing distance travelled and rearing behavior, respectively (Tru Scan^TM^, Photobeam Sensor-E63-22, Coulbourn Instruments). The time spent in the center was also determined. Amphetamine-induced hyperactivity and positive 50-kHz USV were assessed under red light (28 lux) conditions.

- - 1. **Recording and analysis of ultrasonic vocalizations**

UltraSoundGate Condenser CM 16 Microphones (Avisoft Bioacoustics) used for USV recordings were connected via an UltraSoundGate 416H USB audio device (Avisoft Bioacoustics) to a personal computer, where acoustic data were recorded with a sampling rate of 250,000 Hz in 16 bit format (recording range: 0-125 kHz) by Avisoft RECORDER USGH (Avisoft Bioacoustics). The microphones are sensitive to frequencies of 15-180 kHz with a flat frequency response (±6 dB) between 25-140 kHz. For acoustical analysis, recordings were transferred to Avisoft SASLab Pro (version 4.50; Avisoft Bioacoustics). High resolution spectrograms (frequency resolution: 488 Hz; time resolution: 0.512 ms) were obtained through a fast Fourier transformation (512 FFT length, 100% frame, Hamming window and 75% time window overlap). Call detection of isolation-induced 40-kHz USV emitted by pups was provided by an automatic whistle tracking algorithm (maximum frequency change: 1.953 Hz) in combination with a hold-time mechanism (hold time: 20 ms) and post filters (minimum duration: 5 ms; maximum entropy 0.55). Since no USV were detected below 30 kHz, a high-pass filter of 30 kHz was used to reduce background noise outside the relevant frequency band to 0 dB. The accuracy of call detection by the software was verified manually by an experienced observer blind to experimental conditions (detection rate: 98.6-99.5 %, false alarm rate: 0.2-2.8 %). Additional parameters, based on previous USV studies,[25] included peak frequency and peak amplitude, i.e. loudness, which were derived from the average spectrum of the entire call, were determined automatically. Peak amplitude was defined as the point with the highest energy within the spectrum. Peak frequency was defined as the frequency at the location of the peak amplitude within the spectrum. In addition, the extent of frequency modulation, i.e. the difference between the lowest and the highest peak frequency within each call, was measured automatically. Temporal parameters included latency to start calling, total calling time, and call duration. Call detection of pro-social and positive 50-kHz USV emitted by juvenile rats during rough-and-tumble play and adult rats following the administration of amphetamine, respectively, was provided by an experienced observer blind to experimental conditions, who manually counted the numbers of USV in 20 s time bins. USV emitted within a frequency range of 20-32 kHz were considered as 22-kHz USV and USV with peak frequencies higher than 32 kHz as 50-kHz USV.[34] If two 50-kHz USV elements were at least 10 ms apart, two independent 50-kHz USV were counted.[34] Based on previous studies on 50-kHz USV,[34,36] however, additional parameters were determined for positive 50-kHz USV in half of the rats exposed to amphetamine. Additional parameters were determined in the first 20 s of each minute and included total calling time, call duration, peak frequency, peak amplitude, and frequency modulation. Based on peak frequency and shape, 50-kHz USV were further classified into four different subtypes according to the following criteria (for details see refs[34,36]): Flat 50-kHz USV (FLAT): a 50-kHz USV was categorized as FLAT when peak frequency changes within a single call element were equal or lower than 5 kHz. However, the difference between start and end peaks could be higher than 5 kHz, i.e. calls with a flat shape in either upward or downward direction were also considered as FLAT 50-kHz USV. Step 50-kHz USV (STEP): when a fundamental FLAT 50-kHz USV had at least one short flat element overlapped at the start and/or end of the 50-kHz USV. At least one of these short ‘steps’ had to be about 5 kHz higher than the fundamental 50-kHz USV. Trill 50-kHz USV (TRILL): a single call element either with one peak frequency change higher than 5 kHz or with two or more peak frequency changes in opposed directions at least 5 kHz apart (i.e. zigzag-shaped call). Mixed 50-kHz USV (MIXED): Frequency-modulated 50-kHz USV (FM) that did not fall within the previous categories of STEP and TRILL, such as trills with one or more flat and/or step components. Whenever appropriate, STEP, TRILL, and MIXED 50-kHz USV were summed up and simply referred to as FM calls. Of note, aversive 22-kHz USV occurred very rarely and were therefore not included in the analysis.

- 1. **Hippocampal neuronal plasticity and microRNAs**

Rats were deeply anesthetized using isoflurane (Baxter, Unterschleißheim, Germany) and decapitated at PND 312±5 for brain removal. RNA was purified from rat hippocampal tissue (N=12 CONT; N=12 APA) by means of the mirVana miRNA Isolation Kit (Ambion, Carlsbad, CA, USA) and treated with TURBO DNase (Ambion) to remove genomic DNA. As previously reported,[29,37] quantitative real-time PCR (qRT-PCR) was performed with a StepOnePlus 7300 Real Time PCR System (Applied Biosystems, Foster City, CA, USA) using iTaq SybrGreen Supermix with ROX (Bio-Rad Laboratories, Hercules, CA, USA) for mRNAs and pre-miRNAs (primer sequences are available on request) and TaqMan miRNA Assay (Applied Biosystems) for the detection of mature miRNAs. U6 was used as qRT-PCR normalization control and the average of triplicate CT values from each sample was used to calculate the relative RNA amount (2^-∆CT^).

- - 1. **Statistical analysis**

For comparing maternal care between CONT and APA rats, two-way ANOVAs for repeated measurements with the between subject factor paternal age (AGE) and the within subject factor postnatal day (PND) were performed. For analyzing APA effects on sex ratios in litters of both cohorts, Pearson's chi-squared tests were used. Isolation-induced 40-kHz USV and developmental milestones were analyzed by means of three-way ANOVAs for repeated measurements with the between subject factors AGE and SEX and the within subject factor PND, followed by unpaired t-tests when appropriate (p<.05). For analyzing spatial learning and reversal learning, two-way ANOVAs for repeated measurements with the between subject factor AGE and the within subject factor DAY or ARM were used, followed by unpaired t-tests (CONT versus APA) and paired t-tests (previously baited versus not baited and currently baited versus previously baited arms) when appropriate (p<.05). PPI of acoustic startle was compared using two-way ANOVAs for repeated measurements with the between subject factor AGE and the within subject factor pre-pulse (PRE_PULSE). For comparing mRNAs, pre-miRNAs, and mature miRNAs, a two-way ANOVA with the between subject factor AGE and HEMISPHERE was performed. Two-sampled unpaired t-tests were used for all other comparisons between CONT and APA rats. Comparisons of changes over days within CONT and APA rats were made by means of two-sampled paired t-tests. One-sampled t-tests were used for comparing against 50% chance levels in novel object recognition and sucrose preference as well as for comparing PPI of acoustic startle against 100% baseline levels. Data are presented as mean±SEM. A p-value of <.05 was considered statistically significant.

**Supplementary Figure 1: Effect of maternal age on grey matter volume.** Maternal age was linearly correlated with decreased grey matter volume in the right IFG (MNI coordinates: 38; 27; -11, cluster extend 54 voxels, p < .001, unc.).

**Supplementary Figure 2: No evidence for behavioral phenotypes with relevance for depression, schizophrenia or bipolar disorder in APA rats.** (A) and (B) Liquid intake [%] of a sucrose solution with water also being available; chance level is indicated by the dashed line for a 1.0 % and 0.5 % sucrose solution, respectively. (C) Pre-pulse inhibition of acoustic startle response [% of baseline] with the pulse presented alone and in combination with pre-pulses of different intensities (64, 68, 72, and 76) presented before the pulse; baseline acoustic startle response is indicated by the dashed line. (D), (E), and (F) Locomotor activity as assessed by the distance traveled [cm] in an open field following (D) no injection (BASELINE), (E) injection of saline solution (SALINE), or (F) injection of amphetamine solution (2.5mg/kg; AMPHETAMINE). (G), (H), and (I) Emission of positive 50-kHz ultrasonic vocalizations (USV) [n] after (G) no injection (BASELINE), (H) injection of saline solution (SALINE), or (I) injection of amphetamine solution (2.5mg/kg; AMPHETAMINE). (J) Detailed analysis of 50-kHz USV subtypes. Left: Each area represents the number of calls of a given subtype, expressed as the percentage of all 50-kHz USV. Right: Exemplary sonograms of the four 50-kHz USV subtypes analyzed (namely flat, step, trill, and mixed). ^#^p<.050.

**Supplementary Figure 3: No evidence for cognitive deficits in APA rats.** (A) Exploration ratio after a 5 minute exposure to an unfamiliar and a previously explored object. Values above 0.5 indicate a preference towards the novel object; chance discrimination is indicated by the dashed line. (B) Wrong arm entries [%] during the six days of training in the spatial learning period using a radial eight arm maze with three consistently baited arms. (C) Time spent on arms [%/arm] during the 5 minute probe trial that was performed as an additional trial on the last spatial learning day with no food pellets provided. ^#^p<.050.

**Supplementary Figure 4: No evidence for differences in maternal behavior between mothers raising CONT and APA litters.** (A) Frequency of anogenital licking displayed by the mother towards its pups [n/h]; (B) Picture of a rat mother licking one of its pups. (C) Frequency of arched-back nursing events [n/h]. (D) Frequency of the mother being outside the nest [n/h]. (E) Frequency of at least one pup being outside the nest [n/h]. (F) Latency for retrieval of the first pup [s] after pups were scattered around the nest.

SUPPLEMENTARY FIGURE 1


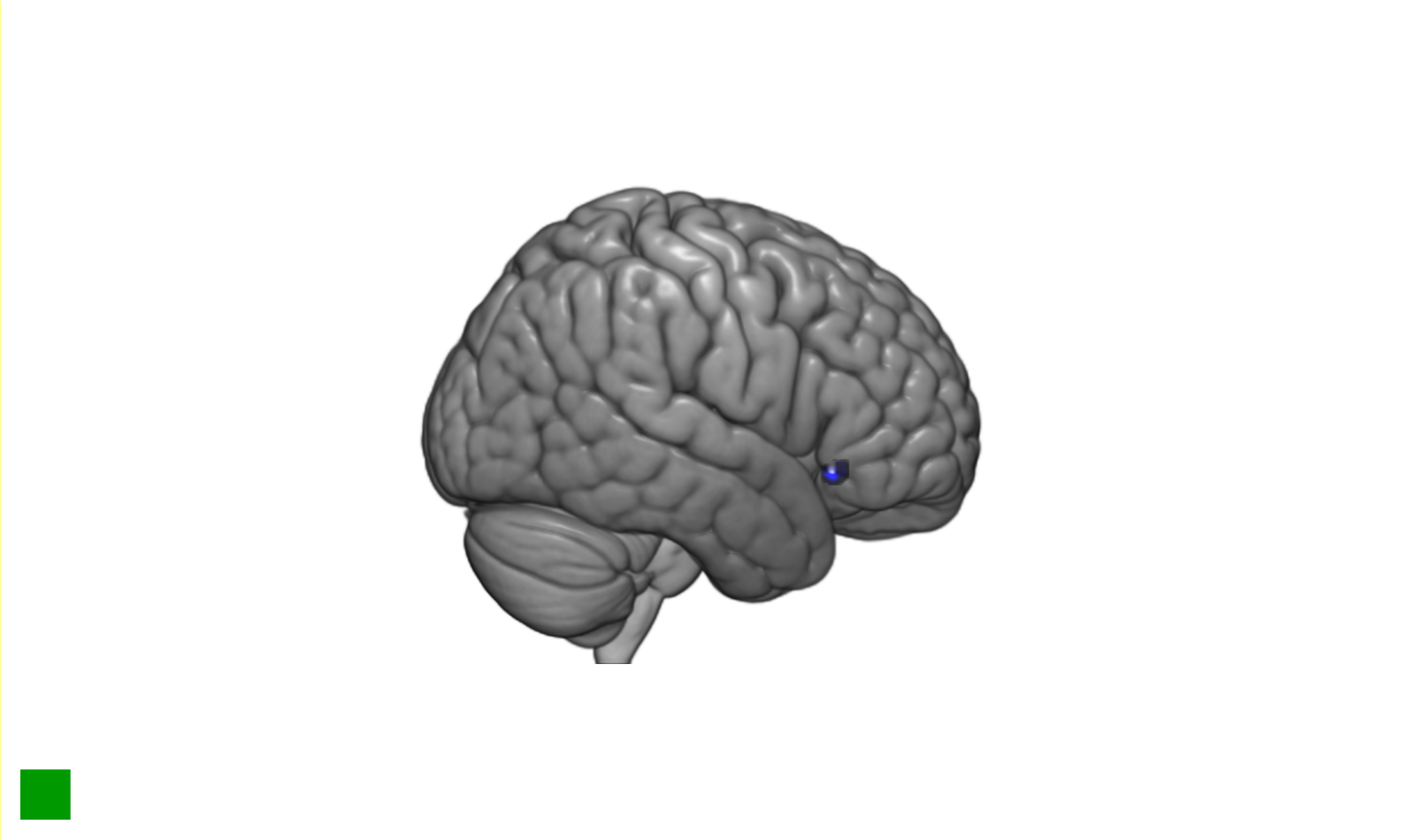


SUPPLEMENTARY FIGURE 2


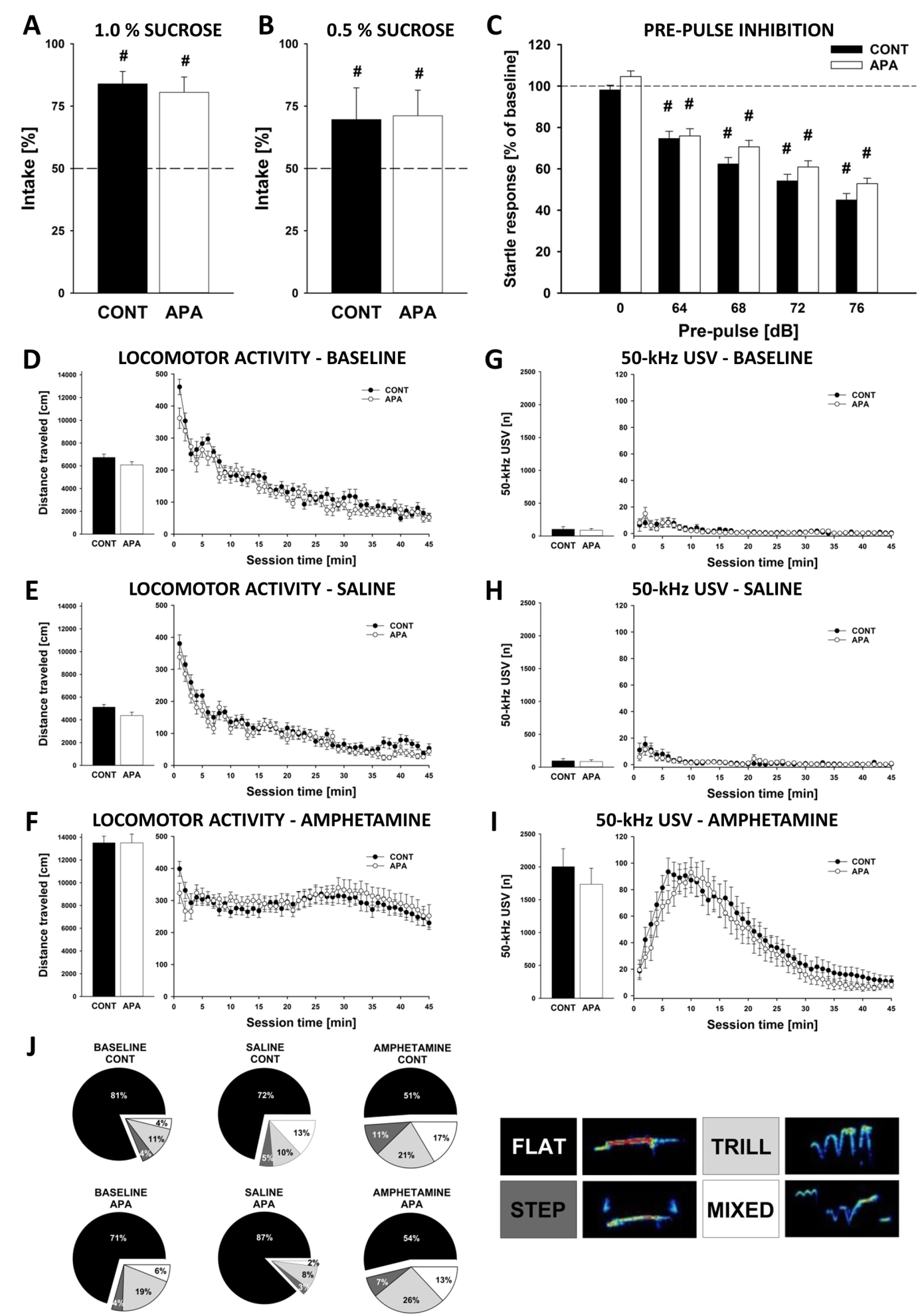


SUPPLEMENTARY FIGURE 3


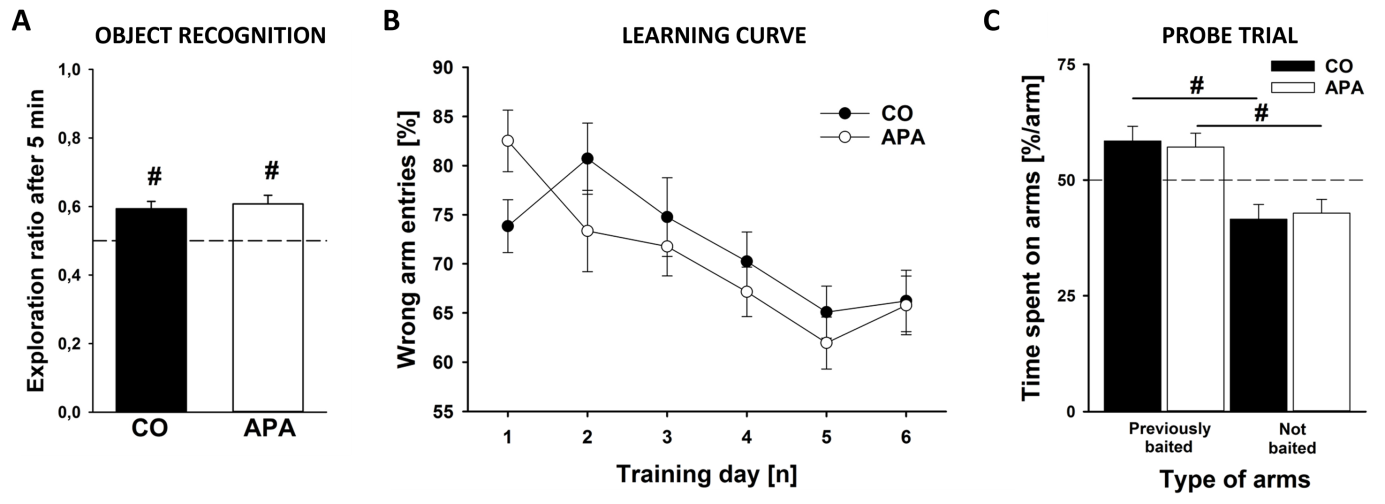


SUPPLEMENTARY FIGURE 4


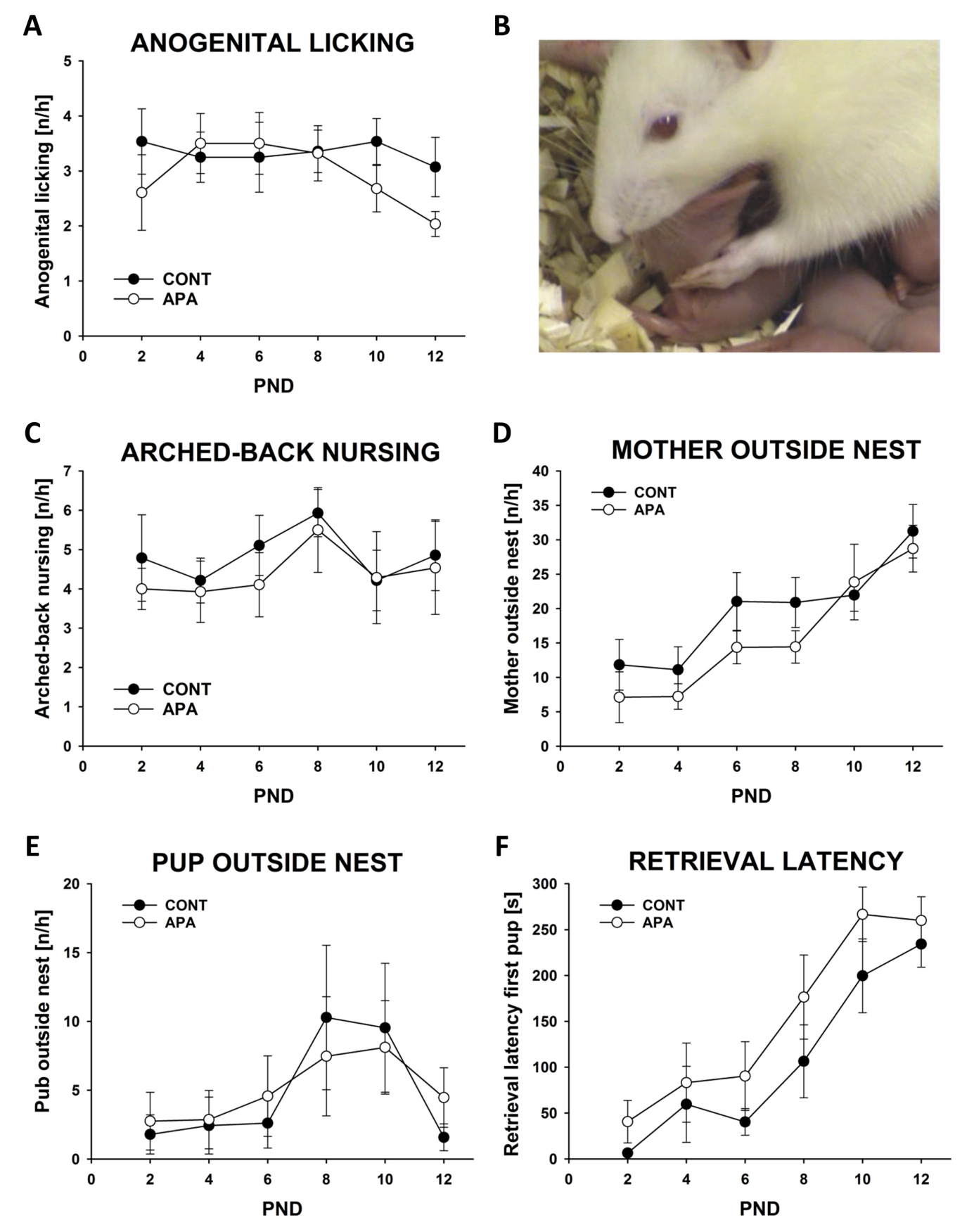


1. Borkenau P, Ostendorf F. NEO-Fünf-Faktoren-Inventar nach Costa und Mc Crae. Göttingen: Hogrefe; 2008.

2. Raine A, Benishay D. The SPQ-B: A Brief Screening Instrument for Schizotypal Personality Disorder. J Pers Disord [Internet]. Guilford Publications Inc.; 1995;9:346–55. Available from: https://doi.org/10.1521/pedi.1995.9.4.346

3. Brickenkamp R. Der Aufmerksamkeits-Belastungstest d2. Göttingen: Hogrefe; 2002.

4. Lehrl S, Triebig G, Fischer B. Multiple choice vocabulary test MWT as a valid and short test to estimate premorbid intelligence. Acta Neurol Scand [Internet]. 1995 [cited 2016 May 22];91:335–45. Available from: http://www.ncbi.nlm.nih.gov/pubmed/7639062

5. Reitan RM. Trail Making Test. Manual for administration and scoring. Tuscon, USA: Reitan Neuropsychology Laboratory; 1979.

6. Aschenbrenner A, Tucha O, Lange K. RWT Regensburger Wortflüssigkeits-Test. Göttingen: Hogrefe; 2000.

7. Gold JM, Carpenter C, Randolph C, Goldberg TE, Weinberger DR. Auditory working memory and Wisconsin Card Sorting Test performance in schizophrenia. Arch Gen Psychiatry. 1997;54:159–65.

8. Wechsler D, Coalson DL, Raiford SE. WAIS-IV: Wechsler adult intelligence scale. Pearson San Antonio, TX; 2008.

9. Wechsler F. Wechsler Memory Scale: Administration and Scoring Manual. San Antonio, TX: Harcourt Brace & Co; 1997.

10. Helmstaedter C, Lendt M, Lux S. Verbaler Lern- und Merkfähigkeitstest. Göttingen: Hogrefe; 2001.

11. Smith SM, Jenkinson M, Johansen-Berg H, Rueckert D, Nichols TE, Mackay CE, et al. Tract-based spatial statistics: voxelwise analysis of multi-subject diffusion data. Neuroimage [Internet]. 2006 [cited 2018 Feb 3];31:1487–505. Available from: http://www.ncbi.nlm.nih.gov/pubmed/16624579

12. Smith SM, Nichols TE. Threshold-free cluster enhancement: addressing problems of smoothing, threshold dependence and localisation in cluster inference. Neuroimage [Internet]. 2009 [cited 2018 Feb 3];44:83–98. Available from: http://linkinghub.elsevier.com/retrieve/pii/S1053811908002978

13. Aryee MJ, Jaffe AE, Corrada-Bravo H, Ladd-Acosta C, Feinberg AP, Hansen KD, et al. Minfi: a flexible and comprehensive Bioconductor package for the analysis of Infinium DNA methylation microarrays. Bioinformatics [Internet]. 2014 [cited 2018 Feb 3];30:1363–9. Available from: https://academic.oup.com/bioinformatics/article-lookup/doi/10.1093/bioinformatics/btu049

14. Ritchie ME, Phipson B, Wu D, Hu Y, Law CW, Shi W, et al. limma powers differential expression analyses for RNA-sequencing and microarray studies. Nucleic Acids Res [Internet]. 2015 [cited 2018 Feb 3];43:e47. Available from: http://academic.oup.com/nar/article/43/7/e47/2414268/limma-powers-differential-expression-analyses-for

15. Du P, Zhang X, Huang C-C, Jafari N, Kibbe WA, Hou L, et al. Comparison of Beta-value and M-value methods for quantifying methylation levels by microarray analysis. BMC Bioinformatics [Internet]. 2010 [cited 2018 Feb 3];11:587. Available from: http://bmcbioinformatics.biomedcentral.com/articles/10.1186/1471-2105-11-587

16. Johnson WE, Li C, Rabinovic A. Adjusting batch effects in microarray expression data using empirical Bayes methods. Biostatistics [Internet]. 2007 [cited 2018 Feb 4];8:118–27. Available from: http://www.ncbi.nlm.nih.gov/pubmed/16632515

17. Leek JT, Johnson WE, Parker HS, Jaffe AE, Storey JD. The sva package for removing batch effects and other unwanted variation in high-throughput experiments. Bioinformatics [Internet]. 2012 [cited 2018 Feb 4];28:882–3. Available from: https://academic.oup.com/bioinformatics/article-lookup/doi/10.1093/bioinformatics/bts034

18. Houseman EA, Accomando WP, Koestler DC, Christensen BC, Marsit CJ, Nelson HH, et al. DNA methylation arrays as surrogate measures of cell mixture distribution. BMC Bioinformatics [Internet]. 2012 [cited 2018 Feb 4];13:86. Available from: http://bmcbioinformatics.biomedcentral.com/articles/10.1186/1471-2105-13-86

19. Witt S, Dukal H, Hohmeyer C, Radosavljevic-Bjelic S, Schendel D, Frank J, et al. Biobank of Psychiatric Diseases Mannheim – BioPsy. Open J Bioresour [Internet]. Ubiquity Press; 2016 [cited 2020 Mar 3];3. Available from: http://openbioresources.metajnl.com/articles/10.5334/ojb.18/

20. Grota LJ, Ader R. Continuous recording of maternal behaviour in Rattus norvegicus. Anim Behav [Internet]. 1969;17:722–9. Available from: http://www.sciencedirect.com/science/article/pii/S0003347269800199

21. Silverman JL, Yang M, Lord C, Crawley JN. Behavioural phenotyping assays for mouse models of autism. Nat Rev Neurosci [Internet]. 2010 [cited 2018 Feb 3];11:490–502. Available from: http://www.nature.com/articles/nrn2851

22. Young JW, Henry BL, Geyer MA. Predictive animal models of mania: hits, misses and future directions. Br J Pharmacol [Internet]. 2011 [cited 2018 Feb 4];164:1263–84. Available from: http://www.ncbi.nlm.nih.gov/pubmed/21410454

23. Powell CM, Miyakawa T. Schizophrenia-relevant behavioral testing in rodent models: a uniquely human disorder? Biol Psychiatry [Internet]. 2006 [cited 2018 Feb 4];59:1198–207. Available from: http://linkinghub.elsevier.com/retrieve/pii/S0006322306005920

24. Cryan JF, Holmes A. The ascent of mouse: advances in modelling human depression and anxiety. Nat Rev Drug Discov [Internet]. 2005 [cited 2018 Feb 4];4:775–90. Available from: http://www.nature.com/articles/nrd1825

25. Wöhr M, Schwarting RKW. Maternal care, isolation-induced infant ultrasonic calling, and their relations to adult anxiety-related behavior in the rat. Behav Neurosci [Internet]. 2008 [cited 2018 Feb 4];122:310–30. Available from: http://doi.apa.org/getdoi.cfm?doi=10.1037/0735-7044.122.2.310

26. Grota LJ, Ader R. Effects of litter size on emotionality, adrenocortical reactivity, and susceptibility to gastric erosions in the rat. Psychol Rep [Internet]. 1969 [cited 2018 Feb 4];24:547–9. Available from: http://journals.sagepub.com/doi/10.2466/pr0.1969.24.2.547

27. Lukas M, Wöhr M. Endogenous vasopressin, innate anxiety, and the emission of pro-social 50-kHz ultrasonic vocalizations during social play behavior in juvenile rats. Psychoneuroendocrinology [Internet]. 2015 [cited 2018 Feb 4];56:35–44. Available from: http://linkinghub.elsevier.com/retrieve/pii/S030645301500092X

28. Schwarting RKW, Jegan N, Wöhr M. Situational factors, conditions and individual variables which can determine ultrasonic vocalizations in male adult Wistar rats. Behav Brain Res [Internet]. 2007 [cited 2018 Feb 4];182:208–22. Available from: http://www.ncbi.nlm.nih.gov/pubmed/17367876

29. Valluy J, Bicker S, Aksoy-Aksel A, Lackinger M, Sumer S, Fiore R, et al. A coding-independent function of an alternative Ube3a transcript during neuronal development. Nat Neurosci [Internet]. 2015 [cited 2018 Feb 4];18:666–73. Available from: http://www.nature.com/articles/nn.3996

30. Bevins RA, Besheer J. Object recognition in rats and mice: a one-trial non-matching-to-sample learning task to study “recognition memory”. Nat Protoc [Internet]. 2006 [cited 2018 Feb 4];1:1306–11. Available from: http://www.nature.com/doifinder/10.1038/nprot.2006.205

31. Görisch J, Schwarting RKW. Wistar rats with high versus low rearing activity differ in radial maze performance. Neurobiol Learn Mem [Internet]. 2006 [cited 2018 Feb 4];86:175–87. Available from: http://linkinghub.elsevier.com/retrieve/pii/S107474270600027X

32. Brosda J, Hayn L, Klein C, Koch M, Meyer C, Schallhorn R, et al. Pharmacological and parametrical investigation of prepulse inhibition of startle and prepulse elicited reactions in Wistar rats. Pharmacol Biochem Behav [Internet]. 2011 [cited 2018 Feb 4];99:22–8. Available from: http://linkinghub.elsevier.com/retrieve/pii/S0091305711000967

33. Rippberger H, van Gaalen MM, Schwarting RKW, Wohr M. Environmental and Pharmacological Modulation of Amphetamine- Induced 50-kHz Ultrasonic Vocalizations in Rats. Curr Neuropharmacol [Internet]. 2015 [cited 2018 Feb 4];13:220–32. Available from: http://www.ncbi.nlm.nih.gov/pubmed/26411764

34. Wöhr M, Rippberger H, Schwarting RKW, van Gaalen MM. Critical involvement of 5-HT2C receptor function in amphetamine-induced 50-kHz ultrasonic vocalizations in rats. Psychopharmacology (Berl) [Internet]. 2015 [cited 2018 Feb 4];232:1817–29. Available from: http://link.springer.com/10.1007/s00213-014-3814-9

35. Natusch C, Schwarting RKW. Using bedding in a test environment critically affects 50-kHz ultrasonic vocalizations in laboratory rats. Pharmacol Biochem Behav [Internet]. 2010 [cited 2018 Feb 4];96:251–9. Available from: http://linkinghub.elsevier.com/retrieve/pii/S0091305710001498

36. Pereira M, Andreatini R, Schwarting RKW, Brenes JC. Amphetamine-induced appetitive 50-kHz calls in rats: a marker of affect in mania? Psychopharmacology (Berl) [Internet]. 2014 [cited 2018 Feb 4];231:2567–77. Available from: http://link.springer.com/10.1007/s00213-013-3413-1

37. Brenes JC, Lackinger M, Höglinger GU, Schratt G, Schwarting RKW, Wöhr M. Differential effects of social and physical environmental enrichment on brain plasticity, cognition, and ultrasonic communication in rats. J Comp Neurol [Internet]. 2016 [cited 2018 Feb 4];524:1586–607. Available from: http://doi.wiley.com/10.1002/cne.23842
